# Systemic nanoparticle delivery of CRISPR-Cas9 ribonucleoproteins for effective tissue specific genome editing

**DOI:** 10.1038/s41467-020-17029-3

**Published:** 2020-06-26

**Authors:** Tuo Wei, Qiang Cheng, Yi-Li Min, Eric N. Olson, Daniel J. Siegwart

**Affiliations:** 1 Department of Biochemistry, Simmons Comprehensive Cancer Center, The University of Texas Southwestern Medical Center, Dallas, TX USA; 2 Department of Molecular Biology, Hamon Center for Regenerative Science and Medicine, The University of Texas Southwestern Medical Center, Dallas, TX USA; 3 Senator Paul D. Wellstone Muscular Dystrophy Cooperative Research Center, The University of Texas Southwestern Medical Center, Dallas, TX USA

**Keywords:** Gene delivery, Nucleic-acid therapeutics, CRISPR-Cas9 genome editing, Biomedical engineering, Drug delivery

## Abstract

CRISPR-Cas9 has emerged as a powerful technology that relies on Cas9/sgRNA ribonucleoprotein complexes (RNPs) to target and edit DNA. However, many therapeutic targets cannot currently be accessed due to the lack of carriers that can deliver RNPs systemically. Here, we report a generalizable methodology that allows engineering of modified lipid nanoparticles to efficiently deliver RNPs into cells and edit tissues including muscle, brain, liver, and lungs. Intravenous injection facilitated tissue-specific, multiplexed editing of six genes in mouse lungs. High carrier potency was leveraged to create organ-specific cancer models in livers and lungs of mice though facile knockout of multiple genes. The developed carriers were also able to deliver RNPs to restore dystrophin expression in DMD mice and significantly decrease serum PCSK9 level in C57BL/6 mice. Application of this generalizable strategy will facilitate broad nanoparticle development for a variety of disease targets amenable to protein delivery and precise gene correction approaches.

## Abstract (web summary)

Therapeutic targets of CRISPR-Cas can often not be accessed due to lack of carriers to deliver RNPs systematically. Here, the authors engineer modified lipid nanoparticles for delivery of gene editing proteins to specific tissues.

## Introduction

The clustered regularly interspaced short-palindromic repeat (CRISPR)-associated protein 9 (CRISPR-Cas9) nuclease system has emerged as a powerful genome editing tool for various biomedical applications^1–9^. The single-guide RNA (sgRNA) recognizes specific target genomic loci and guides the Cas9 nuclease to cleave the recognized DNA sequence. Induced double-stranded breaks are repaired through non-homologous end joining to generate insertions or deletions (termed Indels) or homology-directed repair for gene modifications. Among various strategies^1,10–14^, delivery of Cas9/sgRNA RNPs is an attractive approach because it is the most direct and rapid method for CRISPR-Cas gene editing and has been shown to have less off-target effects and lower immunogenicity^10,15–21^.

Despite this obvious promise, delivery of RNPs is the most challenging approach for non-viral carriers due to the large size of Cas9, the highly negative charge of sgRNAs, and the difficulty of protecting RNPs from degradation or denaturation during the entire formulation and delivery process. To date, several non-viral nanovectors have been reported for in vitro RNP delivery into cells, including DNA nanoclews^22^, cationic lipid nanoparticles and lipoplexes^23–25^, gold-based nanoparticles^26–28^, and zeolitic imidazole frameworks^29^. Most approaches utilize host–guest and electrostatic interactions, often adsorbing RNPs after nanoparticle formation. Consequently, it is difficult to control the size, uniformity, and stability of the resulting formulations, which has limited in vivo applications to local administrations, such as inner ears^23,25^, muscles^26^, brain^30,31^, and tumors^28^. At present, development of stable nanoparticles for systemic delivery of RNPs to targeted organs remains elusive.

**Fig. 1:**
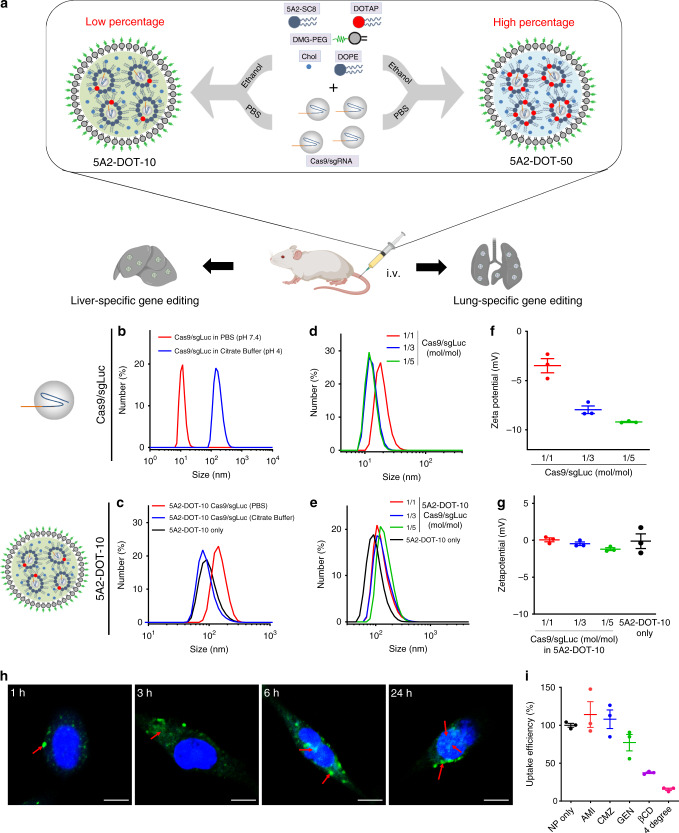
A modular approach was developed to enable systemic nanoparticle delivery of CRISPR-Cas9 RNPs for tissue-specific genome editing. **a** Addition of a permanently cationic supplemental component (e.g., DOTAP) into traditional LNP formulations enabled encapsulation and protection of Cas9/sgRNA complexes using neutral buffers during nanoparticle formation. Precise tuning of the DOTAP percentage mediated tissue-specific gene editing. **b** Size distribution of Cas9/sgLuc RNPs prepared in PBS buffer (pH 7.4) and citrate buffer (pH 4.0). The size increase is likely due to denaturization. **c** Size distribution of 5A2-DOT-10 encapsulating Cas9/sgLuc RNPs prepared in PBS and citrate buffer. 5A2-DOT-10 prepared without RNPs was used as control. **d** Size distribution of Cas9/sgRNA RNPs with Cas9/sgLuc molar ratio of 1/1, 1/3, and 1/5. **e** Size distribution of 5A2-DOT-10 encapsulating Cas9/sgLuc with molar ratio of 1/1, 1/3, and 1/5. **f** Zeta potential of Cas9/sgRNA RNPs showing decreasing charge. Data are presented as mean ± s.e.m. (*n* = 3 biologically independent samples). **g** No significant difference of zeta potential was observed for 5A2-DOT-10 encapsulating Cas9/sgLuc with different molar ratios. Data are presented as mean ± s.e.m. (*n* = 3 biologically independent samples). **h** Time-dependent cellular uptake of 5A2-DOT-10 LNPs encapsulating EGFP-fused Cas9/sgRNAs showing cytoplasmic release and gradual entry into the nucleus (*n* = 3 biologically independent samples). Scale bar: 10 μm. Red arrows show distribution of EGFP-fused Cas9/sgRNAs inside cells. **i** Inhibition of 5A2-DOT-10 LNP uptake was studied using specific endocytosis inhibitors. Amiloride (AMI): inhibitor of macropinocytosis; chlorpromazine (CMZ): inhibitor of clathrin-mediated endocytosis; Genistein (GEN): inhibitor of caveolae-mediated endocytosis; Methyl-β-cyclodextrin (MβCD): lipid rafts-mediated endocytosis; 4 degree: energy-mediated endocytosis. Data are presented as mean ± s.e.m. (*n* = 3 biologically independent samples). Source data are in the Source Data file.

Based on a fundamental understanding of these issues, we developed a generalizable engineering approach that is rationally designed to preserve RNP integrity through inclusion of a permanently cationic lipid in established ionizable lipid nanoparticle (LNP) formulations (Fig. 1a). We show that the supplemental lipid component mediates encapsulation of RNPs with retention of activity and redirects DNA editing to targeted tissues, including the lungs and liver following low-dose intravenous (IV) injection. We further demonstrate that this approach is applicable to three different classes of LNPs, including dendrimer lipid nanoparticles (DLNPs), stable nucleic acid lipid particles (SNALPs), and lipid-like nanoparticles (LLNPs). This led to development of modified lipid nanoparticles capable of rapidly editing the DNA of cells in different tissues. High DLNP potency allowed facile creation of organ-specific cancer models in the livers and lungs of mice. Modified DLNPs also delivered RNPs to restore dystrophin expression in DMD mice and significantly decrease serum PCSK9 level in C57BL/6 mice. Collectively, these results indicate that the generalizable approach developed here is applicable to diverse gene editing areas, including fundamental biology modeling and therapeutic intervention.

## Results

### Development of a methodical strategy to deliver RNPs

Central to our development of a generalizable strategy to load and stabilize RNPs into nanoparticles was the realization that established dilution methods (e.g., ethanol/acidic buffer) commonly used to formulate various lipid- and polymer-based nanoparticles are discordant with delivery of RNPs. We observed that Cas9 RNPs denature in acidic buffer, resulting in an increase of hydrodynamic size from 10 to 150 nm (Fig. 1b). This makes RNP encapsulation into monodisperse nanoparticles difficult if not impossible. We focus here on lipid nanoparticles (LNPs) because they are the most efficacious class of RNA delivery carriers^1,32–34^ in preclinical models and in humans^35^. Among the four components of LNPs [ionizable cationic lipids, zwitterionic phospholipids, cholesterol, and poly(ethylene glycol) (PEG) lipids)], ionizable cationic lipids with pKa around 6.4 are essential for activity because they bind negatively charged RNAs at the pH of mixing (e.g., pH 4 when the amines are protonated), lose charge at neutral pH before cellular uptake, and then acquire charge again as the pH in endosomes decreases to fuse with endosomal membranes and enable release of cargoes to the cytoplasm. However, this critical feature prevents effective encapsulation of cargoes at neutral pH because ionizable cationic lipids are uncharged at neutral pH. To overcome this challenge, we hypothesized that supplemental addition of a cationic lipid that would be positively charged at neutral pH, would allow for encapsulation of RNAs and proteins using neutral buffers (instead of acidic buffers), thus preserving the essential tertiary structure and stability of RNPs (Fig. 1a).

To evaluate this strategy, we selected 5A2-SC8 as the ionizable cationic lipid because 5A2-SC8 LNPs safely delivered short small-interfering RNAs (siRNAs)/microRNAs (miRNAs) and long mRNAs to mice with compromised liver function, including *MYC*-driven liver cancer^36–38^ or genetic knockout of fumarylacetoacetate hydrolase (FAH)^39^. Excitingly, introduction of a permanently cationic lipid (e.g., 1,2-dioleoyl-3-trimethylammonium-propane (DOTAP)) into traditional 4-component 5A2-SC8 LNP formulatons indeed allowed controlled self-assembly to occur by mixing an ethanol solution of lipids with a phosphate-buffered saline (PBS) solution of RNPs (1/3, v/v). We evaluated incorporation of DOTAP from 5 to 60 mole% of total lipids (Supplementary Figs. 1 and 20), which revealed higher levels of gene editing at 10–20% in vitro and formation of stable RNP-loaded nanoparticles with size <200 nm (Supplementary Fig. 2). Initially using sgRNA targeting reporter Luciferase (sgLuc), we observed that the size of LNPs with 10 mole% DOTAP incorporation termed 5A2-DOT-10 (5A2-SC8/DOPE/Chol/DMG-PEG/DOTAP = 15/15/30/3/7 (mol/mol)), prepared using PBS buffer were slightly larger than nanoparticles without RNP loading. Identical LNPs prepared using low pH buffer did not change size, implying that RNPs were not encapsulated (Fig. 1c). To determine the optimal mole ratio between Cas9 protein and sgRNA, we prepared Cas9/sgRNA complexes at 1/1, 1/3, and 1/5 (mol/mol), which decreased RNP size (Fig. 1d) and increased negative charge (Fig. 1f). Encouragingly, the RNP ratio did not alter the size or zeta potential of the resulting LNPs after encapsulation (Fig. 1e, g). The surface charge of all LNPs was neutral, which not only indicates successful encapsulation, but is also important for minimizing in vivo uptake by the immune mononuclear phagocyte system (MPS) system. To further examine whether 5A2-DOT-10 could successfully mediate delivery of RNPs into the nucleus, we tracked LNPs with encapsulated fluorescent EGFP-fused Cas9 protein. Free RNPs alone were unable to enter cells, as no green fluorescence above background was detectable (Supplementary Fig. 3). Strikingly, bright green fluorescence was observed in the cytoplasm of cells after 5A2-DOT-10 treatment for 3 h. EGFP-fused Cas9 proteins were then observed to gradually enter the nucleus within 6 h (Fig. 1h) due to the presence of nuclear localization signals on Cas9. Endocytosis was energy dependent and mainly dependent on lipid rafts, as treatment of MβCD, an inhibitor of lipid raft-based endocytosis, significantly inhibited cellular uptake of nanoparticles (Fig. 1i).

### Highly efficient genome editing was achieved in vitro

**Fig. 2:**
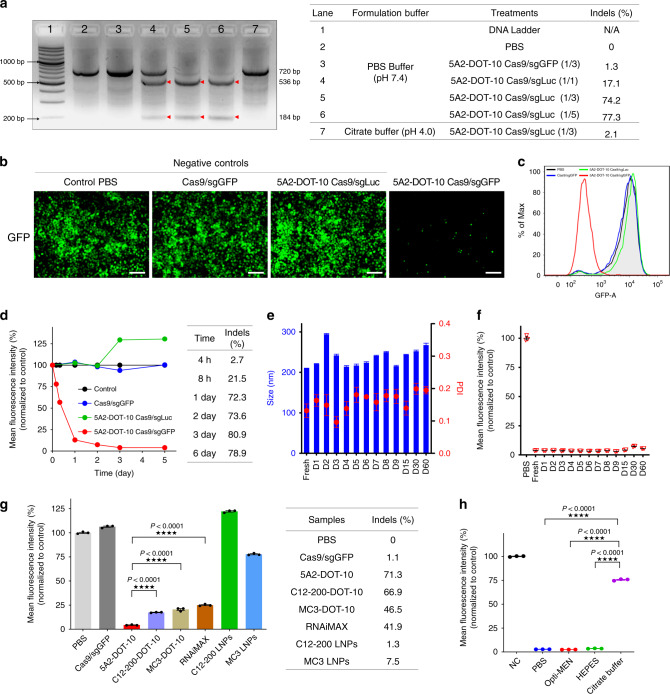
Gene editing occurs quickly and effectively in vitro. **a** T7EI cleavage assay of DNA isolated from HeLa-Luc cells treated with various formulations. Highly effective gene editing was mediated by 5A2-DOT-10 delivering Cas9/sgLuc RNPs (1/3 and 1/5). Red arrows indicate cleavage bands. Indels (%) at *Luc* locus was quantified by Sanger sequencing and TIDE analysis. This experiment was repeated three times independently with similar results. **b** Fluorescence microscopy images of HeLa-GFP cells after treatment with various formulations (*n* = 3 biologically independent samples). Scale bar = 100 μm. 5A2-DOT-10 Cas9/sgGFP treatment significantly decreased GFP fluorescence. **c** Flow cytometry analysis of HeLa-GFP cells after treatment with various formulations. The peak of GFP-positive cells shifted completely to the left only for the 5A2-DOT-10 Cas9/sgGFP group, indicating almost all GFP-positive cells went dark. **d** Time-dependent GFP fluorescence intensity of HeLa-GFP cells after various treatments (mean ± s.e.m., *n* = 3 biologically independent samples). Permanent GFP fluorescence loss was observed with 5A2-DOT-10 Cas9/sgGFP treatment, which was supported by Sanger sequencing and TIDE analysis. **e** 5A2-DOT-10 Cas9/sgGFP LNPs were stored at 4 °C for 2 months. The nanoparticle diameter and PDI was monitored over time (mean ± s.e.m., *n* = 4 biologically independent samples). **f** Periodic treatment of HeLa-GFP cells with stored LNPs showed that no activity was lost, indicating long-term LNP and RNP stability (mean ± s.e.m., *n* = 3 biologically independent samples). **g** Mean fluorescence intensity (%) of HeLa-GFP cells after treatment with Cas9/sgGFP alone, 5A2-DOT-10, C12-200-DOT-10, MC3-DOT-10, C12-200 LNPs, MC3 LNPs, and Cas9/sgGFP-loaded RNAiMAX (mean ± s.e.m., *n* = 3 biologically independent samples). The GFP fluorescence significantly decreased after treated with all three DOTAP-modified formulations. TIDE analysis of Sanger sequencing data further confirmed the highest gene editing efficiency was with 5A2-DOT-10 LNPs. **h** Mean fluorescence intensity (%) of Hela-GFP cells after treatment with 5A2-DOT-10 formulated with Cas9/sgGFP in different buffers. Neutral buffer was required for RNP encapsulation and delivery (mean ± s.e.m., *n* = 3 biologically independent samples). One-way ANOVA followed by Dunnett’s multiple comparison test was used to determine the significance in **g** and **h**. (*P < 0.05; **P < 0.01; ***P < 0.001; ****P < 0.0001). Source data are in the Source Data file.

To quantify gene editing efficacy, we employed HeLa-Luc and HeLa-GFP reporter cells. Examining different Cas9/sgLuc ratios, gene editing was higher at 1/3 and 1/5 than at 1/1 (Fig. 2a). The result of a T7 Endonuclease I (T7EI) assay demonstrated that most all target DNA bands (720 bp) were cut into two cleavage bands (536 bp and 184 bp) at ratios of 1/3 and 1/5. No cleavage bands were observed with control treatment groups. Therefore, we fixed the molar ratio of Cas9/sgRNA at 1/3 for all subsequent studies. To test our hypothesis that neutral pH buffer is required to encapsulate RNPs with preservation of Cas9 function, we also evaluated the gene editing efficiency of 5A2-DOT-10 prepared using pH 4 citrate buffer. No cleavage bands were observed (Fig. 2a) by T7EI. This result was further confirmed by Sanger sequencing, providing additional evidence that the conventional acid-based formulation methods could not produce efficacious NPs. Switching to GFP-expressing cells, 5A2-DOT-10 encapsulating Cas9/sgGFP induced Indels into GFP DNA and knocked out nearly all GFP expression. Control groups exhibited similar fluorescence intensity with PBS-treated cells (Fig. 2b), which was confirmed by flow cytometry (Fig. 2c and Supplementary Fig. 4). Permanent gene editing was apparent by an indefinite loss of GFP in growing cells for at least 5 days (Fig. 2d and Supplementary Fig. 5a). With an eye towards eventual clinical translation, we monitored RNP-loaded 5A2-DOT-10 stability at 4 °C for 2 months. LNPs did not change size and remained uniform (PDI < 0.2) (Fig. 2e and Supplementary Fig. 5b). Continual testing of 5A2-DOT-10 nanoparticles revealed constant gene editing activity, even after 60 days storage (Fig. 2f). These results are very promising for future clinical translation.

### The approach is applicable to other LNP types and neutral buffers

The strategy of adding a permanently cationic lipid into classical 4-component LNPs to achieve efficient RNP delivery was not limited to the dendrimer-based ionizable lipid, 5A2-SC8. To prove this, we included supplemental DOTAP into nanoformulations prepared using other classes of ionizable materials: the well-known DLin-MC3-DMA lipid used in FDA-approved Onpattro^35^ and the C12-200 lipidoid (Supplementary Fig. 6a, b). Even though they have very different chemical structures compared to 5A2-SC8 (Supplementary Fig. 6c), all DOTAP-modified nanoparticles could efficiently edit cells whereas previously established C12-200 or MC3 formulations without DOTAP showed low editing efficiency (Fig. 2g and Supplementary Fig. 6d). 5A2-DOT-10 also achieved higher editing efficiency than the positive control RNAiMAX. As 5A2-DOT-10 LNPs were more efficacious than MC3-DOT-10 and C12-200-DOT-10, we performed all subsequent experiments using 5A2-SC8. In addition to DOTAP, we also introduced other cationic lipids, including DDAB and EPC, into LNP formulations (Supplementary Fig. 6e–g). The results were similar for all three cationic lipids with different chemical structures (Supplementary Fig. 6h). These results indicate that this strategy is universal for ionizable cationic lipid nanoparticles (DLNPs, LLNPs, SNALPs) and for other cationic lipids that are positively charged at pH 7.4. As our methodology allowed adjustment of the FDA-approved Onpattro formulation to enable delivery of RNPs, this approach offers new directions for clinically translatable treatment of human diseases.

A key to successful RNP delivery is replacement of the standard acidic buffer with PBS buffer to maintain protein stability. To test if our methodology is compatible with other neutral buffers, we formulated LNPs in PBS, Opti-MEM media, and HEPES. Formulations prepared in citrate buffer (pH 4) were used as a control (Supplementary Fig. 6i). Significant and equivalent gene editing was achieved using LNPs prepared in all three neutral buffer conditions, but not in acidic buffer (Fig. 2h and Supplementary Fig. 6j). Sanger sequencing results were consistent with that shown by flow cytometry (Supplementary Fig. 6k). This result further confirmed that neutral buffer was critical for RNP delivery.

### Effective multiplexed genome editing was achieved in vivo

**Fig. 3:**
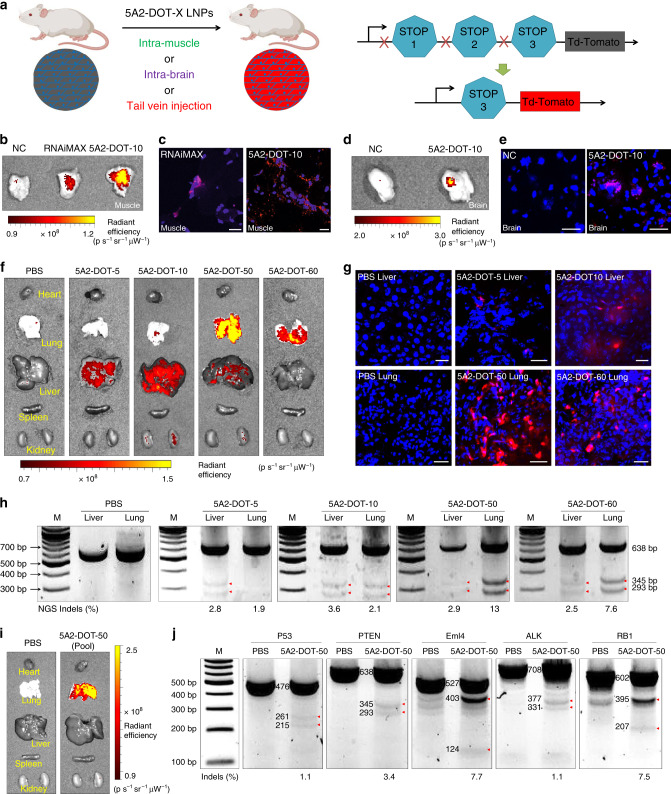
Highly efficient multiplexed genome editing was achieved in vivo. **a** Schematic illustration shows how delivery of Cas9/sgTOM RNPs activates Td-Tom expression in Td-Tomato transgenic mice. 5A2-DOT-X LNPs were injected into Td-Tom mice locally (via intra-muscle or intra-brain injections) and systemically (via IV injection through tail vein). In vivo imaging of Td-Tom mice after intra-muscle (1 mg kg^−1^ sgTOM) (**b**) or intra-brain (0.15 mg kg^−1^ sgTOM) (**d**) injection of 5A2-DOT-10 Cas9/sgTOM showed bright red fluorescence in the leg muscle or brain tissue (respectively). Successful CRISPR-Cas gene editing was further confirmed by confocal imaging of **c** muscle and **e** brain tissue sections. Scale bar: 20 μm. 5A2-DOT-10 enabled higher gene editing efficiency than positive control RNAiMAX, which has previously been used for local RNP injections. **f** In vivo imaging of Td-Tom mice after intravenous (IV) injection of 5A2-DOT-X Cas9/sgTOM LNPs with different molar percentages of DOTAP. Td-Tom fluorescence, as a downstream readout of DNA editing, showed that low DOTAP percentages facilitated liver editing while high DOTAP percentages facilitated lung editing (1.5 mg kg^−1^ sgTOM, IV). **g** Successful CRISPR-Cas gene editing was further confirmed by confocal imaging. Scale bar: 20 μm. **h** The T7EI cleavage assay was performed on DNA isolated from liver and lung tissues after systemic IV treatment with 5A2-DOT-5, 5A2-DOT-10, 5A2-DOT-50, and 5A2-DOT-60 encapsulating Cas9/sgPTEN. Red arrows indicate cleavage bands generated. Indels (%) was calculated using next generation sequencing (NGS) of DNA isolated from harvested tissues. **i** 5A2-DOT-50 LNPs containing pooled sgRNAs for six targets (sgTOM, sgP53, sgPTEN, sgEml4, sgALK, and sgRB1) (5A2-DOT-50-Pool) were administered to td-Tom mice IV at total RNA dose of 2 mg kg^−1^ (0.33 mg kg^−1^ each sgRNA). Gene editing at the *TOM* locus was confirmed by in vivo imaging and **j** editing of the other five loci was confirmed using the T7EI assay on lung tissues. Indels percentages were measured using Sanger sequencing and TIDE analysis. Red arrows indicate cleavage bands generated. Data of **c**, **e**, **g**, **h**, and **j** were repeated three times independently with similar results.

To examine in vivo gene editing, we delivered 5A2-DOT-10 encapsulating Cas9/sgTOM complexes to the Td-Tomato mouse (Ai9 mouse) model (Fig. 3a). The genetically engineered Ai9 mouse harbors three repeated stop cassettes (SV40 polyA sequences) that prevent expression of the tdTomato fluorescent protein. CRISPR-mediated deletion of two repeat cassettes is sufficient to activate downstream tdTom expression^31^. This allows facile determination of successfully gene edited cells. We injected 5A2-DOT-10 LNPs loaded with Cas9/sgTOM RNPs into the left leg of mice by intramuscular injection at dose of 1 mg kg^−1^ sgTOM. Owing to previous use for gene editing via local injection^23^, RNAiMAX complexed with Cas9/sgTOM RNPs was used for comparison. Higher Td-Tom fluorescence was observed in the muscle treated with 5A2-DOT-10 than in mice treated with RNAiMAX (Fig. 3b). Imaging of tissue sections further confirmed gene editing producing brighter red fluorescence in the 5A2-DOT-10 treatment group (Fig. 3c). Next, we injected 5A2-DOT-10 into the brains of Td-Tom mice (0.15 mg kg^−1^ of sgTOM). Again, bright red signal was observed near the injection site, confirming editing of mouse brains (Fig. 3d, e). We envision future utility for correction of Central Nervous System diseases.

We next evaluated whether the improved stability and efficacy of 5A2-DOT-10 could mediate successful systemic gene editing in tissues. In parallel to this report, we have explored a variety of additional 5th lipids and formulated a complete methodology for organ-specific delivery of mRNA^40^. We found that systematic adjustment of the molar percentage and chemical identity of supplemental molecules precisely modulates tissue biodistribution and release of mRNA in targeted cells. Building on this recent advance, we prepared LNPs with different molar percentages of DOTAP (5–60%) and delivered RNPs to Td-Tom mice IV (1.5 mg kg^−1^ of sgTOM). Excitingly, Td-Tom fluorescence was observed exclusively in the liver 7 days following injection of 5A2-DOT-5. Increasing the incorporated DOTAP percentage from 5 to 60% resulted in gradual fluorescence (CRISPR-guided gene editing) from liver to lung. 5A2-DOT-60 enabled mainly lung editing (Fig. 3f and Supplementary Fig. 7). These results indicate that deep tissue editing can be achieved in a tissue-specific manner by adjusting the inner lipid component chemistry and molar ratios. Tissue-specific editing was further confirmed by confocal imaging of tissue sections (Fig. 3g). We next examined editing of an endogenous target, *PTEN*, by systemically injecting LNPs encapsulating Cas9/sgPTEN RNPs into wild-type C57BL/6 mice. Clear T7EI cleavage bands were only detected in liver for 5A2-DOT-5 treated mice and in the lungs for 5A2-DOT-50 and 5A2-DOT-60 treated mice (Fig. 3h and Supplementary Fig. 20). Next generation deep sequencing (NGS) performed on DNA isolated from mouse livers and lungs further confirmed high gene-editing efficacy (Fig. 3h). In addition, we utilized the Cas-OFFinder prediction webtool to identify the top 10 ranking sgPTEN off-target sites. We then amplified these ten potential off-target sites from 5A2-DOT-50 Cas9/sgPTEN RNPs treated lungs and measured CRISPR-Cas editing using T7EI assay. No off-target cutting was observed at any of these off-target sites (Supplementary Figs. 8 and 20).

To evaluate whether it is possible to edit multiple genes in vivo, we encapsulated Cas9 protein and six different sgRNAs into 5A2-DOT-50. sgTOM, sgP53, sgPTEN, sgEml4, sgALK, and sgRB1 were loaded into Cas9 proteins. We then treated Td-Tom mice with 5A2-DOT-50 (Pool) by tail vein injection (0.33 mg kg^−1^ of each sgRNA). After 1 week, bright Td-Tom fluorescence was detected in the lungs, indicating gene editing of TOM (Fig. 3i). Clear T7EI cleavage bands were observed at all other five genome loci, demonstrating 5A2-DOT-50 LNPs were able to edit multiple genes in the lungs effectively (Fig. 3j and Supplementary Fig. 20) at low doses (0.33 mg kg^−1^ sgRNA). We note that sgRNAs with end modifications of the first and last three nucleotides were used herein to enhance sgRNA stability and reproducibility (Supplementary Fig. 9)^13,41^. Reports have shown that precise modifications to additional nucleotides can increase in vivo gene editing two- to fourfold compared to end-modified sgRNAs^13,42^, suggesting that the editing efficiencies reported herein could be higher with further sgRNA optimization. Nevertheless, the high potency and tissue specificity of 5A2-DOT-50 allowed for multiplexed editing in the lungs with one injection.

### Generation of complex mouse models via intravenous injection

Animal models are traditionally generated by transgenesis or gene engineering in embryonic stem cells, which is time consuming and costly. Direct mutation of tumor- and other disease-related genes in adult mice using CRISPR-Cas provides a feasible approach for rapid generation of models. This has only been accomplished using costly lentiviruses that must be engineered for each target and by hydrodynamic injection into the liver^5,8^. Since mutation of multiple genes is typically required to generate functional cancer models, the development of an inexpensive and effective non-viral nanoparticle-based approach for multiplexing is highly desirable. Since 5A2-DOT-X LNPs are potent, can simultaneously edit multiple targets, can be administered repeatedly, and provide tissue specificity, they provide a compelling path to generate a wide variety of animal models.

**Fig. 4:**
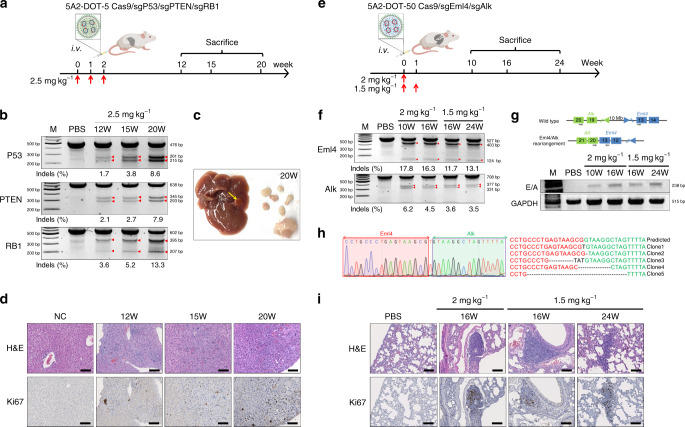
5A2-DOT-X LNPs simplify generation of complex mouse models. **a** To create an in situ liver-specific cancer model, 5A2-DOT-5 LNPs encapsulating Cas9/sgP53/sgPTEN/sgRB1 RNPs were injected into adult C57BL/6 mice weekly (three injections, 2.5 mg kg^−1^ total sgRNA, IV, *n* = 4). After 12, 15, and 20 weeks, mice were sacrificed and livers were collected to analyze tumor generation. **b** T7EI cleavage results from genomic DNA extracted from livers confirmed gene editing occurred at all three loci. Red arrows indicate cleavage bands. Indel percentages shown under gel images were measured by Sanger sequencing and TIDE analysis. **c** Representative photograph of a mouse liver containing tumors excised 20 weeks after injection. **d** H&E and Ki67 staining further confirmed progressive tumor formation. Higher tumor proliferation biomarker Ki67 expression was detected in tumor lesions. Scale bar = 100 μm. **e** To create an in situ lung-specific cancer model, 5A2-DOT-50 LNPs encapsulating Cas9/sgEml4/sgAlk RNPs were injected into adult C57BL/6 mice once (2 mg kg^−1^) or twice (1.5 mg kg^−1^ weekly for 2 weeks) (IV, *n* = 5). After 10, 16, and 24 weeks, mice were sacrificed and lungs were collected to analyze tumor generation. **f** T7EI cleavage results from genomic DNA extracted from lungs confirmed gene editing occurred at loci of *Eml4* and *Alk*. Red arrows indicate cleavage bands. Indel percentages shown under gel images were measured by Sanger sequencing and TIDE analysis. **g** PCR amplicons of Eml4-Alk rearrangements were also detected in all lungs treated with 5A2-DOT-50 LNPs. **h** Eml4-Alk rearrangements were further confirmed by sub-cloning and DNA sequencing. **i** H&E and Ki67 staining further confirmed progressive tumor formation. Higher tumor proliferation biomarker Ki67 expression was detected in lung tumor lesions. Scale bar = 100 μm. Data of **b**, **d**, **f**, **g**, and **i** were repeated three times independently with similar results.

To prove this, we employed 5A2-DOT-5 to knockout three tumor suppressor genes (P53, PTEN, and RB1) selectively in the liver. These genes have been identified in many human cancers, including liver. We treated C57BL/6 mice with weekly IV injections of 2.5 mg kg^−1^ total sgRNA for 3 weeks and detected the gene editing efficiency in mice livers (Fig. 4a). We observed clear cleavage bands at all three gene loci after treatments of 2, 12, 15, and 20 weeks by T7EI assay (Fig. 4b and Supplementary Figs. 10, 11, 20). The cleavage bands were much brighter as time progressed, indicating tumor growth. When we sacrificed mice at 15 weeks and 20 weeks, we found visible tumors on the liver, together with several metastatic tumors in the abdominal cavities (Fig. 4b and Supplementary Fig. 12). We also detected the tumor generation by H&E staining and IHC staining targeting tumor proliferation biomarker Ki67 (Fig. 4d and Supplementary Fig. 13) at various time points.

To generate a challenging lung cancer mouse model, we focused on the Eml4-Alk chromosomal rearrangement (Supplementary Fig. 14), which is a complex mutation found in many solid human tumors, especially non-small cell lung cancers^8,43^. The Eml4-Alk fusion protein generated after rearrangement between Eml4 and Alk promotes cancer development. Exploiting the high potency and lung-targeting specificity of 5A2-DOT-50, we injected once (at dose of 2 mg kg^−1^ of total sgRNA) or twice (at dose of 1.5 mg kg^−1^ of total sgRNA, weekly) IV and evaluated the tumor generation process (Fig. 4e). Indel generation was detectable at all examined time points from extracted lung DNA from mice in both groups (Fig. 4f and Supplementary Figs. 15 and 20). More importantly, clear gene rearrangement bands were detected in the lungs of 5A2-DOT-50-treated mice, confirming successfully generated chromosomal rearrangements (Fig. 4g and Supplementary Figs. 15 and 20). The sequencing results after sub-cloning of these PCR amplicons further confirmed the Eml4-Alk rearrangements (Fig. 4h and Supplementary Fig. 15). We observed several tumor lesions in the lungs after 16 weeks and 24 weeks from H&E staining and Ki67 staining (Fig. 4i and Supplementary Figs. 16 and 17). These results show that a single injection of 5A2-DOT-50 LNPs could successfully generate chromosomal rearrangements and lead to lung tumor generation in adult mice. These LNPs are therefore positioned to greatly accelerate in situ creation of a variety of disease models.

### Effective gene editing was achieved in therapeutic models

**Fig. 5:**
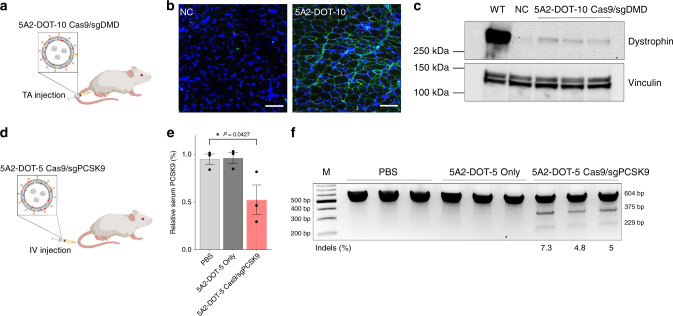
5A2-DOT-X LNPs achieve CRISPR-Cas-based gene editing in therapeutic models. **a** To restore dystrophin expression, 5A2-DOT-10 LNPs encapsulating Cas9/sgDMD RNPs were injected into TA muscles of DMD exon 44 deletion mice weekly (three injections, 1 mg kg^−1^ sgDMD, *n* = 3). Three weeks after the last injection, TA muscles were collected to detect expression of dystrophin protein. **b** Immunofluorescence images indicated that 5A2-DOT-10 LNPs treatment successfully corrected dystrophin gene and restored the expression of dystrophin proteins in TA muscles. 5A2-DOT-10 LNPs nanoparticle only treatment was used as negative control (NC). Scale bar = 100 μm. Data was repeated two times independently with similar results. **c** Western blot analysis further confirmed the expression of dystrophin protein in the 5A2-DOT-10 LNPs encapsulating Cas9/sgDMD RNPs treatment group. 4.2% of dystrophin protein was restored. NC: 5A2-DOT-10 LNPs nanoparticle only; WT wild-type group. **d** To knockout the *PCSK9* gene in mouse liver, 5A2-DOT-5 LNPs encapsulating Cas9/sgPCSK9 RNPs were administered to adult C57BL/6 mice via tail vein injection weekly (three injections, 2.5 mg kg^−1^ sgPCSK9, IV, *n* = 3). One week after the last injection, mouse serum and livers were collected for analyses. **e** The relative PCSK9 level in the serum was significantly decreased in 5A2-DOT-5 LNPs encapsulating Cas9/sgPCSK9 RNPs treatment group, detected using PCSK9 Elisa Kit. Data are presented as mean ± s.e.m. (*n* = 3 biologically independent animals). One-way ANOVA followed by Dunnett’s multiple comparison test was used to determine the significance of data. (*P < 0.05). **f** T7EI cleavage results from genomic DNA extracted from mice livers confirmed gene editing occurred at the *PCSK9* locus. Red arrows indicate cleavage bands. Indel percentages shown under the gel image were measured by Sanger sequencing and TIDE analysis. Source data are in the Source Data file.

In addition to demonstrating utility of 5A2-DOT-X LNPs for creation of animal models, we next aimed to examine the potential for therapeutic intervention. To demonstrate disease therapy, we evaluated 5A2-DOT-X in two therapeutic mouse models. First, we utilized the DMD exon 44 deletion mouse model (ΔEx44 DMD mice), which has recently been shown to recapitulate Duchenne muscular dystrophy (DMD)^44^. Deletion of exon 44 leads to splicing of exon 43 and 45, disrupting the open-reading frame of dystrophin and introducing a premature termination codon. The reading frame can be restored by using CRISPR-Cas9 gene editing to skip or reframe exon 45, which allows splicing between exons 43 and 46 and restoration of functional dystrophin expression^44^. For this, we injected 5A2-DOT-10 LNPs encapsulating Cas9/sgDMD RNPs into Tibialis Anterior (TA) muscles of ΔEx44 DMD mice weekly (three injections, 1 mg kg^−1^ sgRNA) and detected the expression of dystrophin protein in TA muscles 3 weeks after the last injection (Fig. 5a). The expression of dystrophin in TA muscles was successfully restored after treatment with 5A2-DOT-10 LNPs encapsulating Cas9/sgDMD RNPs, demonstrated by immunofluorescence (Fig. 5b) and western blot analysis (Fig. 5c and Supplementary Fig. 20). Quantitate analysis of the western blot result demonstrated that 4.2% of dystrophin protein was restored.

There has recently been increasing interest in using gene editing to knockout genes related to cardiovascular disease as potential long-lasting treatment for chronic and deadly diseases^45^. Among these, the *PCSK9* gene is one of the most attractive drug targets for treating hypercholesterolemia^46,47^. To do this, we injected 5A2-DOT-5 LNPs encapsulating Cas9/sgPCSK9 RNPs into C57BL/6 mice via tail vein injection (three injections, 2.5 mg kg^−1^ sgRNA) and quantified the PCSK9 level in mouse serum and liver tissue (Fig. 5d). IV injection of 5A2-DOT-5 LNPs encapsulating Cas9/sgPCSK9 RNPs significantly decreased PCSK9 protein levels in both serum (Fig. 5e) and liver tissue (Supplementary Figs. 18 and 20). T7EI assay results further confirmed indel generation at the *PCSK9* gene loci in 5A2-DOT-5 LNPs encapsulating Cas9/sgPCSK9 RNPs treated group. (Fig. 5f and Supplementary Fig. 20). These results demonstrate that 5A2-DOT-X LNPs have therapeutic potential for treating diseases.

## Discussion

The modular strategy described here provided a generalizable approach for tissue-specific gene editing via systemic delivery of RNPs. We discovered that incorporation of a permanently cationic lipid into classic LNP formulations facilitated encapsulation of Cas9 RNPs using neutral buffers, which protected and preserved Cas9 function. By adjusting the molecular components and ratios, we achieved tissue-specific gene editing selectively in the livers and lungs of mice following systemic injection. Excitingly, the lung-targeting 5A2-DOT-50 LNPs showed high editing efficiency. The ability of 5A2-DOT LNPs to target multiple genes and create complex animal models in situ has wide applications for protein function discovery, exploration of biological mechanisms, and disease treatment, especially since this can be accomplished with simple IV injections to standard mice of any age. 5A2-DOT LNPs could effectively deliver other proteins (such as ovalbumin) into cell cytoplasm (Supplementary Fig. 19), indicating its significance for other types of protein delivery targeting immunotherapy or protein replacement. The general methodology could also be applied to other delivery systems, including FDA-approved DLin-MC3-DMA SNALPs, further establishing potential translation. The described modular RNP delivery strategy will guide rational design of tissue-specific genomic engineering for a wide variety of preclinical and clinical carriers. We are currently exploring this technology for precise gene correction of various genetic diseases.

## Methods

### Materials

5A2-SC8^36^ and C12-200^48^ were synthesized and purified by following published protocols. DLin-MC3-DMA^49^ was purchased from MedKoo Biosciences. 1,2-dioleoyl-sn-glycero-3-phosphoethanolamine (DOPE), 1,2-dioleoyl-3-trimethylammonium-propane (DOTAP), dimethyldioctadecylammonium (DDAB), 1,2-dimyristoyl-sn-glycero-3-ethylphosphocholine (EPC), 1,2-dioleoyl-sn-glycero-3-phosphoethanolamine (DOPE), and 1,2-distearoyl-sn-glycero-3-phosphocholine (DSPC) were purchased from Avanti Polar Lipids. Cholesterol was purchased from Sigma-Aldrich. 1,2-Dimyristoyl-sn-glycerol-methoxy(poly((ethylene glycol) MW 2000) (DMG-PEG2000) was purchased from NOF America Corporation. The ONE-Glo+Tox Luciferase Reporter assay kit was purchased from Promega Corporation. Pur-A-Lyzer Midi Dialysis Kits (WMCO, 3.5 kDa) were purchased from Sigma-Aldrich. 4’,6-Diamidino-2-phenylindole dihydrochloride (DAPI), Hoechst 33342, DLS Ultramicro cuvettes, Lipofectamine RNAiMAX Transfection Reagent, and Lab-Tek chambered cover glass units were purchased from Thermo Fisher Scientific. Cas9 protein and Ki67 monoclonal antibody was purchased from Thermofisher. Monoclonal anti-dystrophin antibody (D8168) and monoclonal anti-vinculin antibody (V9131) were purchased from Sigma-Aldrich. GenCrispr NLS Cas9-EGFP Nuclease was purchased from GenScript. Modified sgRNAs (Supplementary Table 1) were purchased from Synthego. All primers (Supplementary Table 2) were synthesized by Integrated DNA Technologies (IDT).

### Cas9-sgRNA complex preparation

Separate solutions of Cas9 proteins and sgRNAs in the notated buffers were mixed together at equal volumes. After mixing, the RNPs were allowed to form over 5 min of incubation at room temperature for full Cas9/sgRNA complex self-assembly. The mole ratios of Cas9 protein to sgRNA used were 1/1, 1/3, and 1/5.

### Synthesis and purification of 5A2-SC8

First, to a 20 mL vail was equipped with a stir bar was added tetraethylenepentamine (5A2, 4.0 g, 1.0 equiv.), 2-(acryloyoxy)ethyl methacrylate (AEMA, 20.4 g, 5.25 equiv.), and butylated hydroxyltoluene (BHT, 838 mg, 0.18 equiv.). The resulting reaction mixture was stirred at 50 °C at 500 r.p.m. for 24 h under N_2_. The crude product was purified by flash column chromatography (silica, 30 to 50% acetone/hexanes with 3% triethylamine) to achieve the 5A2-G1 (5.2 g, 19%). Rf = 0.15 (50% acetone/hexanes with 3% triethylamine, silica). Second, to a 20 mL vail was equipped with a stir bar was added 5A2-G1 (3.9 g, 1.0 equiv.), dimethylphenylphosphine (DMPP, 193 μL, 0.45 equiv.), and 1-octanethiol (SC8, 4.71 mL, 6*1.5 equiv.). The resulting reaction mixture was stirred at 55 °C at 500 r.p.m. for 48 h under N_2_. The crude product was dissolved in a minimal amount of CH_2_Cl_2_ and purified by flash column chromatography (neutral Al_2_O_3_, 20 to 100% of ethyl acetate/hexanes) to yield 5A2-SC8 (4.2 g, 64%). Rf = 0.2 (70% ethyl acetate/hexanes, neutral Al_2_O_3_). HRMS Calc. for C_110_H_203_N_5_O_24_S_6_:2170.31, found: 2170.29.

### Synthesis and purification of C12-200

The synthetic protocol was based on a previous report^48^. First, N1-(2-(4-(2-aminoethyl)piperazin-1-yl)ethyl)ethane-1,2-diamine was synthesized by catalytic hydrogenation. 2-(4-(2-((cyanomethyl)amino)ethyl)piperazin-1-yl)acetonitrile (1.0 g) was hydrogenated with H_2_ (g) at 60 psi along with Raney-Nickel 2400 (5 mL slurry, 2.5 g), 25% NH_4_OH (28 mL), and ethanol at r.t. for 23 h. Following workup, 0.98 g N1-(2-(4-(2-aminoethyl)piperazin-1-yl)ethyl)ethane-1,2-diamine was obtained (85.6% yield). Next, N1-(2-(4-(2-aminoethyl)piperazin-1-yl)ethyl)ethane-1,2-diamine was reacted neat with 1,2-epoxydodecane (4/1, mole) in a glass vial at 90 °C for 2.5 days. The crude product was purified by flash column chromatography (silica, CH_2_Cl_2_ to 75∶22∶3 CH_2_Cl_2_/MeOH/NH_4_OH (*aq*)) to yield C12-200. HRMS Calc. for C_70_H_146_N_5_O_5_:1137, found: 1138.

### Optimized nanoparticle formulations and characterization

Ionizable cationic lipids (5A2-SC8, C12-200, or DLin-MC3-DMA)^36,48,49^, zwitterionic lipids (DOPE or DSPC), cholesterol, DMG-PEG, and permanently cationic lipids (DOTAP, DDAB, or EPC) were dissolved in ethanol at given molar ratios. Cas9/sgRNA complexes were dissolved in 1× PBS buffer. The Cas9/sgRNA RNP complexes solution in PBS buffer was pipette mixed rapidly into the lipids solution in ethanol at a volume ratio of 3:1 (Cas9/sgRNA RNPs:lipids, v/v), such that the weight ratio of total lipids to sgRNA was 40:1 (wt), then incubated for 15 min at room temperature. Afterwards, the fresh formulations were directly characterized and used for in vitro assays. For animal experiments, the formulations were dialyzed (Pur-A-Lyzer Midi Dialysis Kits, WMCO 3.5 kDa) against 1× PBS for 1 h to remove ethanol before topical injections (intra-muscle or intra-brain injection) or systemic injection (intravenous injection). The size distribution and zeta potential of nanoformulations were measured using Zetasizer (version 7.13, Malvern Panalytical; He-Ne Laser, *λ* = 632 nm; detection angle = 173°).

### RNAiMAX formulations

For preparation of RNAiMAX complexing RNPs, the Cas9/sgRNA complex was prepared in Opti-MEM and mixed gently with lipofectamine RNAiMAX transfection reagent diluted in Opti-MEM (at dose of 1 μL RNAiMAX per μg sgRNA). The mixture solution was incubated at room temperature for 30 min to complete the complexation.

### Standard LNP formulations

For preparation of C12-200 and MC3 LNPs encapsulating RNPs, Cas9/sgRNA RNP complexes solution in citrate buffer (pH 4.0) was pipette mixed rapidly into the lipids solution in ethanol at a volume ratio of 3:1 (Cas9/sgRNA RNPs: total lipids, v/v), such that the weight ratio of total lipids to sgRNA was 40:1 (wt/wt), then incubated for 15 min at room temperature. The molar ratio of C12-200/DOPE/Chol/DMG-PEG was 35/16/46.5/2.5 for C12-200 LNPs; the molar ratio of DLin-MC3-DMA/DSPC/Chol/DMG-PEG was 50/10/38.5/1.5 for MC3 LNPs.

### Cellular uptake and uptake mechanism

To examine cellular uptake, HeLa-Luc cells were seeded into Lab-Tek Chambered Coverglass (eight wells) at a density of 2 × 10^4^ cells per well, and incubated at 37 °C overnight. Then, the old media was replaced with 150 μL of fresh Dulbecco's modified Eagle Medium (DMEM) containing 10% fetal bovine serum, and treated with 50 μL of 5A2-DOT-10 encapsulating Cas9-EGFP/sgLuc RNPs (9 nM of sgRNA per well). At 1, 3, 6, and 24 h after treatment, cells were washed three times with PBS and stained with Hoechst (0.1 mg mL^−1^) for 15 min at 37 °C, then imaged by confocal microscopy (Zeiss LSM 700) and data were analyzed using ZEN 2010 software version 6.0.62 (Carl Zeiss MicroImaging GmbH).

To examine the uptake mechanism, assays of specific inhibition on endocytosis pathways were evaluated using Hela-Luc cells. 5A2-DOT-10 only treatment was used as a control. HeLa-Luc cells were seeded at a density of 5 × 10^5^ cells per well in 12-well plates and incubated in DMEM complete medium for 24 h. The cells were then washed with PBS and followed by pre-incubating at 37 °C for 1 h with one of the following endocytosis inhibitors dissolved in Opti-MEM: 20 μM chlorpromazine (CMZ, an inhibitor of clathrin-mediated endocytosis), 2 mM Amiloride (AMI, an inhibitor of macropinocytosis), 200 μM Genistein (GEN, an inhibitor of caveolae-mediated endocytosis), 5 mM methyl-β-cyclodextrin (MβCD, an inhibitor of lipid rafts-mediated endocytosis). Next, the medium was removed and replaced with complete DMEM medium containing 5A2-DOT-10 Cas9/sgLuc (24 nM sgLuc) for another 30 min. After that, the medium was removed and the cells were washed three times with PBS. The cells were then collected and analyzed by flow cytometry. All experiments were carried out in triplicate. Here, Cas9-EGFP protein was used to formulate Cas9/sgLuc complex. To evaluate whether it is energy dependent endocytosis, the cells were also pre-incubated under 4 °C for 1 h, and then treated with complete DMEM medium containing 5A2-DOT-10 Cas9/sgLuc (24 nM of sgLuc) for another 30 min before analysis by flow cytometry.

### T7EI assay to detect genomic editing

For in vitro genomic DNA editing analysis, HeLa-Luc cells were seeded into 12-well plates at a cell density of 1.5 × 10^5^ cells/well and incubated overnight. Then, different nanoformulations containing 24 nM of sgRNA were added to cells. After 3 days, the cells were collected, washed, and re-suspended in 50 μL of 1 × passive lysis buffer (Promega) together with 2 μL of proteinase K (Thermofisher). Afterwards, a lysis PCR program (65 °C for 15 min, 95 °C for 10 min) was run to obtain cell lysates. The targeted genomic loci were then amplified using the following PCR amplification program (95 °C for 5 min; (95 °C for 30 s; 60–64 °C for 30 s; 72 °C for 1 min) for 40 cycles; 72 °C for 7 min and then keep at 4 °C). Cell lysates were used as DNA templates. The amplicons were then purified using PCR purification kits (Qiagen) and 200 ng of the purified DNA was added to 19 μL of annealing reaction containing 1 × NEBuffer 2. Then the PCR products were annealed in a thermocycler using the following conditions (95 °C for 5 min, then the mixture was cooled from 95 to 85 °C with Ramp Rate of −2 °C per second, following 85 to 25 °C with Ramp Rate of −0.1 °C per second, then keep at 4 °C) to form heretoduplex DNA. Afterwards, 1 μL of T7EI (NEB) was added and incubated at 37 °C for 15 min. The cleavage reaction was then stopped by adding 1.5 μL of 0.25 M EDTA. Next, the digested DNA was analyzed using 2.5% agarose gel electrophoresis. All primers used for T7EI assay are listed in Supplementary Table 2.

For in vivo genomic DNA editing analysis, genomic DNA was extracted from tissues using PureLink Genomic DNA Mini Kit (Invitrogen) according to manufacturer’s instructions. Subsequently, the aforementioned procedures were followed as described above for T7EI detection.

### Sanger sequencing to quantify genome editing

Purified PCR amplicons together with their forward primers were sequenced by The McDermott Center Sequencing core facility in UT Southwestern Medical Center. The sequencing data was analyzed using Tracking of Indels by Decomposition (TIDE) (https://tide.deskgen.com/). Sanger sequencing/TIDE analysis was performed at the Luc, GFP, TOM, PTEN, P53, RB1, Eml4, Alk, and PCSK9 loci.

### In vitro gene editing in Hela-GFP cells

HeLa-GFP reporter cells were cultured in DMEM containing 10% FBS and 1% penicillin/streptomycin at 37 °C/5% CO_2_. For the experiments, HeLa-GFP cells were seeded into 12-well plates at a cell density of 1.5 × 10^5^ cells per well and incubated overnight. Then, the medium was replaced with 0.5 mL of fresh complete DMEM and 100 μL of nanoparticle dispersion were added (the final concentration of sgRNA was fixed at 24 nM). Three days after treatment, the cells were imaged using Keyence microscope (BZ-X Analyzer software version 1.0.0, Keyence Corporation). For the flow cytometry analysis, the cells were collected, washed with PBS, re-suspended in PBS, and measured using a BD Analyzers LSRFortessa SORP (version 8.0.1, BD Biosciences). The data of flow cytometry were analyzed using FLOWJO software version 7.6 (FLOWJO).

### Stability of 5A2-DOT-10 Cas9-sgGFP

To measure stability, we prepared 5A2-DOT-10 LNPs encapsulating Cas9/sgGFP RNP complexes and stored them at 4 °C for 2 months. The size and PDI of these nanoparticles were tested after storing for different times and their gene editing efficiency were also evaluated in HeLa-GFP cells by adding nanoparticles (24 nM sgRNA dose) and quantifying gene editing after 3 days. For each time point, an aliquot of stored 5A2-DOT-10 LNPs encapsulating Cas9/sgGFP RNPs was taken and analyzed (size, PDI, efficacy).

### Animal experiments

All animal experiments were approved by the Institution Animal Care and Use Committees of The University of Texas Southwestern Medical Center and were consistent with local, state, and federal regulations as applicable. Mice were housed in a barrier facility with a 12-h light/dark cycle and maintained on standard chow (2916 Teklad Global). C57BL/6 mice were obtained from the UTSW Mouse Breeding Core Facility. B6.Cg-*Gt(ROSA)26Sor*^*tm9(CAG-tdTomato)Hze*^/J mice (also known as Ai9 or Ai9(RCL-tdT) mice) were obtained from The Jackson Laboratory (007909) and bred to maintain homozygous expression of the Cre reporter allele that has a loxP-flanked STOP cassette preventing transcription of a CAG promoter-driven red fluorescent tdTomato protein. Following Cas9/sgRNA RNPs-mediated gene editing, Ai9 mice will express tdTomato fluorescence. Ai9 mice are congenic on the C57BL/6J genetic background. ΔEx44 DMD mice were provided by Prof. Eric Olson’s lab^44^.

### In vivo gene editing

For gene editing in muscles, Td-Tomato mice were injected with 5A2-DOT-10 LNPs encapsulating Cas9/sgTOM RNP complexes at dose of 1 mg kg^−1^ of sgTOM in the left leg by intra-muscle injection. RNAiMAX encapsulating Cas9/sgTOM RNP complexes was used as positive control. After treatment for 7 days, the muscle tissues of all treatment groups were collected and imaged using an IVIS Lumina system (Perkin Elmer). Afterwards, the muscle tissues were embedded in optimal cutting temperature (OCT) compound and cut into 10 μm slices. The sections were fixed with 4% Paraformaldehyde (Thermo Fisher Scientific) for 20 min, washed three times using PBS buffer. Afterwards, one drop of ProLong Gold Mountant with DAPI (Thermo Fisher Scientific) was applied onto each slide. A coverslip was placed, and the slides were imaged by confocal microscopy (Zeiss LSM 700) and data were analyzed using ZEN 2010 software version 6.0.62 (Carl Zeiss MicroImaging GmbH). For gene editing in brain, Td-Tomato mice were injected with 5A2-DOT-10 LNPs encapsulating Cas9/sgTOM RNP complexes at dose of 0.15 mg kg^−1^ of sgTOM by intra-brain injection. After treatment for 6 days, the brains were excised and imaged using IVIS Lumina system (version 4.4.0.0, Caliper Life Sciences). Frozen sections of brains were prepared as the protocol mentioned above and imaged by confocal microscopy.

For gene editing by IV injection, mice were treated with 5A2-DOT-X LNPs containing different percentage of DOTAP by tail vein injection. At the sacrifice point, all organs were collected for further analyses.

### In vivo gene editing in ΔEx44 DMD mice

Fifty microliters of 5A2-DOT-10 encapsulating sgDMD was injected into the Tibialis Anterior (TA) muscle of P12 male ΔEx44 DMD mice (at dose of 1 mg kg^−1^ of sgDMD) three times (once per week). Three weeks after the last injection, the TA muscles were removed for western blot (dilution of anti-dystrophin antibody at 1:1000 and dilution of anti-vinculin antibody at 1:5000) and immunofluorescence analyses (*n* = 3 biologically independent animals). 5A2-DOT-10 nanoparticle only treatment group was used as negative control. Quantification of restored dystrophin expression was analyzed using Image J (1.50b Java 1.8.0_60 (64-bit), National Institutes of Health, USA) based on western blot result.

### In vivo PCSK9 gene editing in C57BL/6 mice

5A2-DOT-5 encapsulating sgPCSK9 was injected into C57BL/6 mice by tail vein injection (at dose of 2.5 mg kg^−1^ of sgPCSK9) three times (once per week). One week after the last injection, mouse serum was collected to detect serum PCSK9 level using a PCSK9 Elisa Kit (ab215538, Abcam). The mice were then sacrificed, and liver tissues were collected to detect gene editing at the *PCSK9* gene locus by T7EI assay, Sanger sequencing and western Blot. PBS treatment group and 5A2-DOT-5 nanoparticle only treatment group were used as negative controls (*n* = 3).

### Next generation sequencing

The PCR amplicons at *PTEN* locus were amplified using genome DNA extracted from tissues treated with different 5A2-DOT-X nanoformulations. Then these samples were mailed to GENEWIZ Company to detect gene editing efficiencies using next-generation deep sequencing (NGS).

### PCR for Eml4-Alk rearrangements

The in vivo Eml4-Alk rearrangements were tested by nested PCR^43^. For the first round PCR, 40 ng of genomic DNA was used as template with PCR program of 95 °C for 5 min; (95 °C for 30 s; 64 °C for 30 s; 72 °C for 30 s) for 18 cycles; 72 °C for 7 min and then keep at 4 °C. For the second round PCR, 1 μL of the first round PCR product (100 dilutions) was used for PCR reactions (95 °C for 5 min; (95 °C for 30 s; 68 °C for 30 s; 72 °C for 30 s) for 30 cycles; 72 °C for 7 min and then keep at 4 °C. Primers used in the PCR reactions are listed in Supplementary Table 2. PCR amplified targeting gene *GAPDH* was used as internal control.

### Measurement of off-target effects

Top-10 potential off-target sites were predicted using Cas-OFFinder webtool and these sites were amplified by PCR, then analyzed using T7EI assay. Relative primer information is listed in Supplementary Table 2.

### H&E staining and immunohistochemistry (IHC)

Briefly, 10% formalin solution fixed tissues were embedded in paraffin, sectioned and H&E stained by the Molecular Pathology Core at UTSW. The 4 μm sections were performed in the standard fashion and detected with Elite ABC Kit and DAB Substrate (Vector Laboratories) for IHC. These slides were scanned using NANOZOOMER (NDP.scan software version 3.1.9, Hamamatsu) and analyzed using NDP.view 2 software (version 2.7.25, Hamamatsu).

### Display items

The images of mice (Figs. 1a, 3a, 4a, e, 5a, d), syringes (Figs. 1a, 4a, e, 5a, d), and organs (Figs. 1a, 4a, e) were created with BioRender.com.

### Statistical analyses

Statistical analyses were performed by one-way ANOVA followed by Dunnett’s multiple comparison test, using GraphPad Prism software, version 7.04 (GraphPad Software, USA). A *P-*value < 0.05 was considered statistically significant.

### Reporting summary

Further information on research design is available in the Nature Research Reporting Summary linked to this article.

## Supplementary information

Supplementary Information.pdf

Reporting Summary

## Data Availability

The source data for the Figures along with the Supplementary Figures presented in this paper are available in the Source Data file. Sequencing data is available from the Sequence Read Archive under accession code PRJNA634601. All other relevant data that support all findings within this paper are available from the corresponding author upon request. Source data are provided with this paper.

## Source data

Source Data
