# Supplementary material for: Systemic nanoparticle delivery of CRISPR-Cas9 ribonucleoproteins for effective tissue specific genome editing
Source: Nat Commun. 2020 Jun 26;11:3232. doi: 10.1038/s41467-020-17029-3 (PMC7320157; doi:10.1038/s41467-020-17029-3)
Supplement: Supplementary file 1 — Supplementary Information.pdf [file 41467_2020_17029_MOESM1_ESM.pdf]

Supplementary Information

Systemic nanoparticle delivery of CRISPR-Cas9 ribonucleoproteins for effective tissue specific genome editing

Tuo Wei, Qiang Cheng, Yi-Li Min, Eric N. Olson, and Daniel J. Siegwart*

Supplementary Figures

a

Name	Molar Ratios					Molar Percentage (%)					Total Lipids/ sgRNA (wt/wt)
	5A2-SC8	DOPE	Chol	DMG-PEG	DOTAP	5A2-SC8	DOPE	Chol	DMG-PEG	DOTAP	
5A2-DOT-5	15	15	30	3	3.3	22.6	22.6	45.2	4.5	5	40
5A2-DOT-10	15	15	30	3	7	21.4	21.4	42.8	4.3	10	40
5A2-DOT-20	15	15	30	3	16	19.1	19.1	38.1	3.8	20.3	40
5A2-DOT-30	15	15	30	3	27	16.7	16.7	33.3	3.3	30	40
5A2-DOT-40	15	15	30	3	42	14.3	14.3	28.6	2.9	40	40
5A2-DOT-50	15	15	30	3	63	11.9	11.9	23.8	2.4	50	40
5A2-DOT-60	15	15	30	3	95	9.5	9.5	19.1	1.9	60.1	40

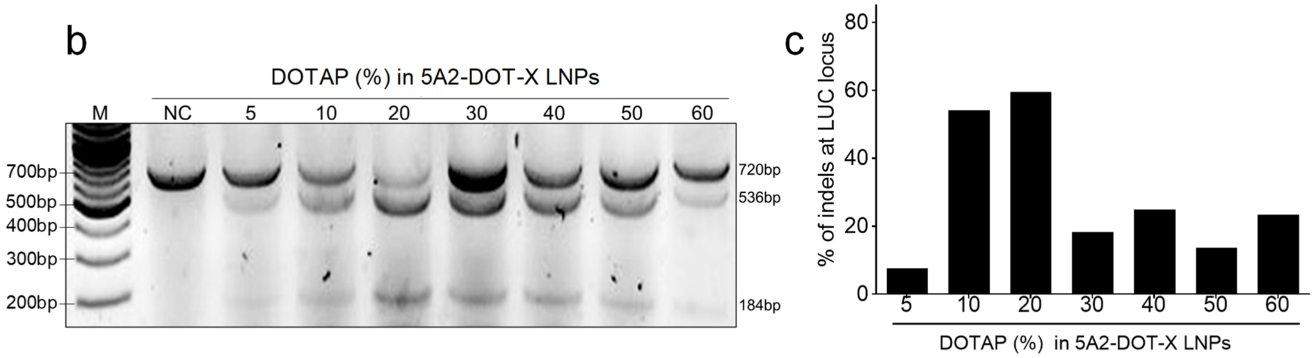

Supplementary Figure 1. Gene editing of 5A2-DOT LNPs incorporating different percentages of DOTAP in Hela-Luc cells. a, Table of 5A2-DOT-X LNPs showing the molar ratios and percentages used to formulate 5A2-DOT-5 (5 mole % DOTAP), 5A2-DOT-10, 5A2-DOT-20, 5A2-DOT-30, 5A2-DOT-40, 5A2-DOT-50, and 5A2-DOT-60 (60 mole % DOTAP) LNPs. A total lipids/sgRNA ratio of 40:1 (wt.) was used for all LNPs. **b,** Gene editing in HeLa-Luc cells following treatment with different 5A2-DOT-X Cas9/sgLuc RNP formulations was detected using the T7EI assay. **c,** Gene editing was analyzed using Sanger sequencing and TIDE analysis.

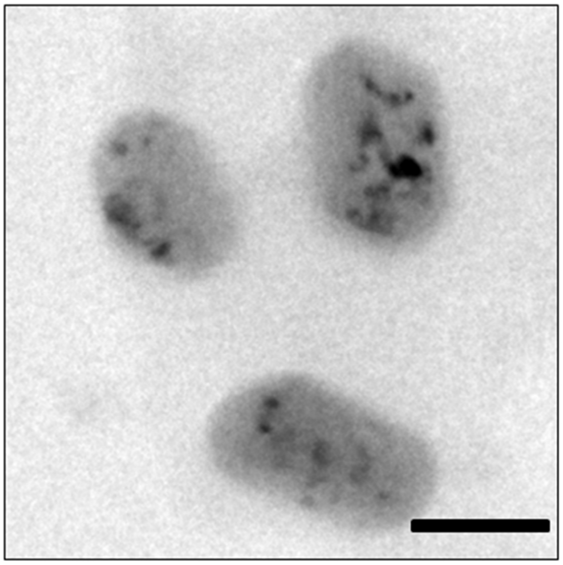

Supplementary Figure 2. Representative TEM images of 5A2-DOT-10 encapsulating Cas9/sgLuc RNP complexes with molar ratio of 1/3. 5A2-DOT-10 Cas9/sgLuc was prepared at total lipid concentration of 2 mg/mL in PBS buffer. 3 uL of the nanoparticle solutions was dropped onto carbon TEM grids and allowed to deposit for 1 min before blotting with filter paper. Then the TEM grids were imaged using Transmission Electron Microscopy (FEI Tecnai G2 Spirit Biotwin) (n=3 independent samples). Scale bar: 100 nm.

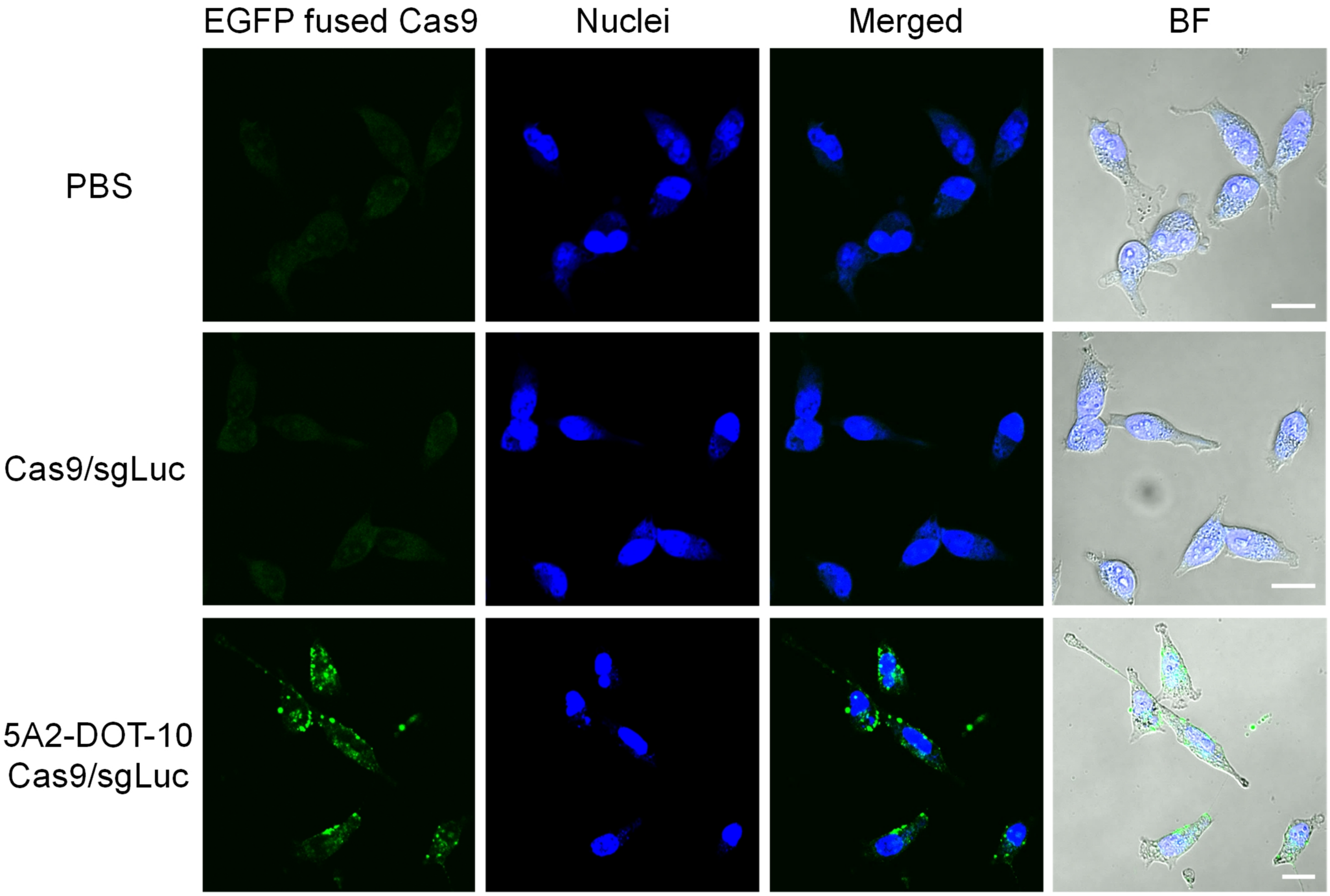

Supplementary Figure 3. **Confocal images showing cellular uptake of 5A2-DOT-10 Cas9/sgLuc in Hela-Luc cells.** Cas9-EGFP fusion protein was used to track the subcellular distribution of Cas9/sgRNA complexes. 20 hours following treatment, the free Cas9/sgLuc complexes exhibited no detectable green fluorescence above background (PBS) inside cells, while bright green signals were detected after treated with 5A2-DOT-10. Scale bar: 20 mm. The data are representative of 3 biologically independent samples (n=3).

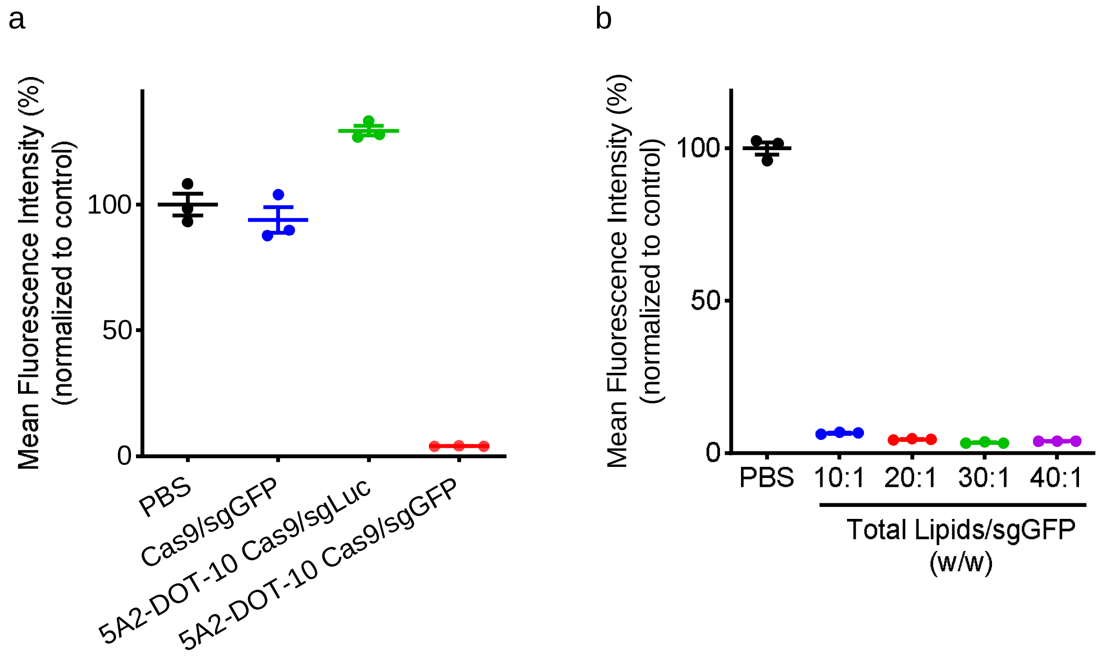

Supplementary Figure 4. Gene editing of different nanoformulations in Hela-GFP cells. a, Mean fluorescence intensity (%) of Hela-GFP cells after treatment with Cas9/sgGFP alone, 5A2-DOT-10 Cas9/sgLuc, and 5A2-DOT-10 Cas9/sgGFP (at total lipids/sgGFP weight ratio of 40:1). **b,** Mean fluorescence intensity (%) of Hela-GFP cells after treatment with 5A2-DOT-10 Cas9/sgGFP prepared with total lipids/sgGFP weight ratio at 10:1, 20:1, 30:1 and 40:1. Data are presented as mean ± s.e.m. (n= 3 biologically independent samples).

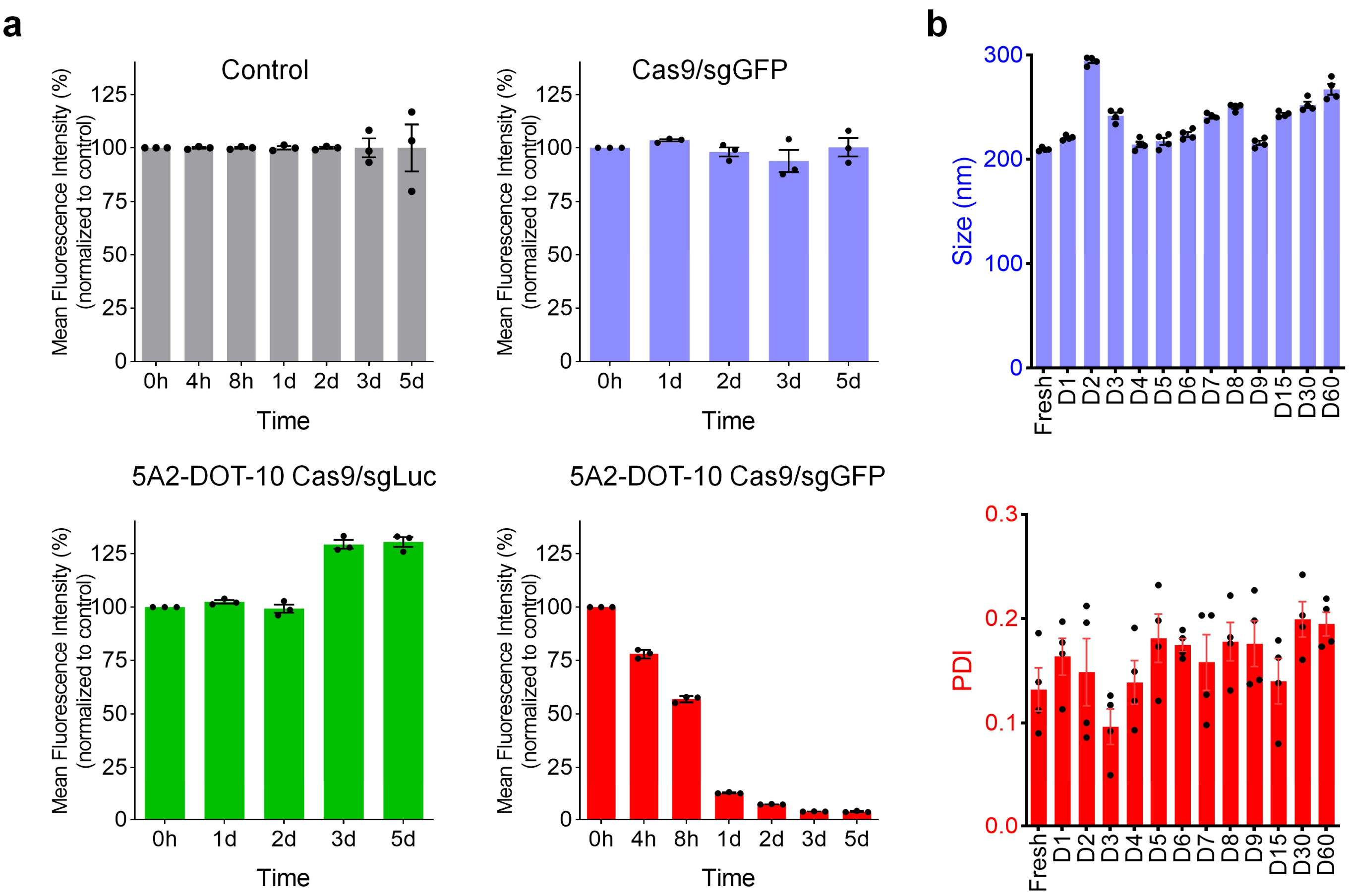

Supplementary Figure 5. Gene editing occurs quickly and effectively in vitro. a, Time-dependent GFP fluorescence intensity of HeLa-GFP cells after various treatments detected by flow cytometry. Permanent GFP fluorescence loss was observed with 5A2-DOT-10 Cas9/sgGFP treatment. Data are presented as mean ± s.e.m. (n= 3 biologically independent samples). **b,** 5A2-DOT-10 Cas9/sgGFP LNPs were stored at 4 °C for 2 months. The nanoparticle diameter and PDI were monitored over time. Data are presented as mean ± s.e.m. (n= 4 biologically independent samples). Please note that Fig. 2d and 2e have been reproduced above in Supplementary Figure 5 to assemble relevant data together for enhanced clarity.

a

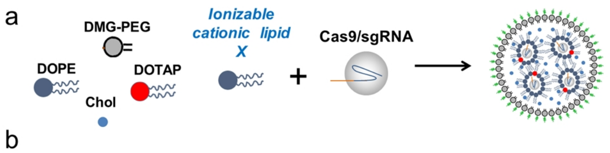

b

Name	Molar Ratios					Molar Percentage (%)					Lipids/ sgRNA (wt/wt)
	X[a]	DOPE DSPC	Chol	DMG-PEG	DOTAP	X[a]	DOPE DSPC	Chol	DMG-PEG	DOTAP	
5A2-DOT-10	15	15	30	3	7	21.4	21.4	42.9	4.3	10	40
C12-DOT-10	35	16	46.5	2.5	11.11	31.5	14.4	41.9	2.3	10	40
MC3-DOT-10	50	10	38.5	1.5	11.11	45	9	34.7	1.4	10	40

[a] X represents 5A2-SC8, C12-200 and DLin-MC3-DMA.

c

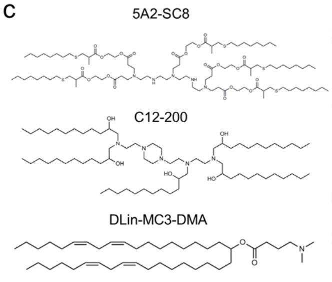

d

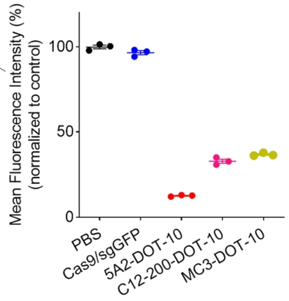

e

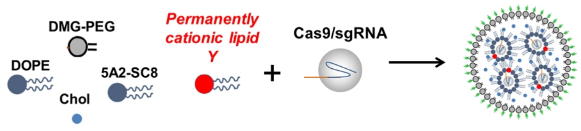

f

Name	Molar Ratios					Molar Percentage (%)					Lipids/ sgRNA (wt/wt)
	5A2-SC8	DOPE	Chol	DMG-PEG	Y[b]	5A2-SC8	DOPE	Chol	DMG-PEG	Y[b]	
DOT-10	15	15	30	3	7	21.4	21.4	42.9	4.3	10	40
DDAB-10	15	15	30	3	7	21.4	21.4	42.9	4.3	10	40
EPC-10	15	15	30	3	7	21.4	21.4	42.9	4.3	10	40

[b] Y represents DOTAP, DDAB and EPC.

g

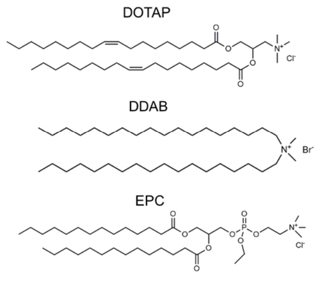

h

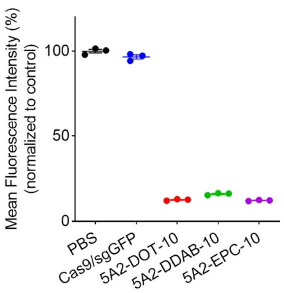

i

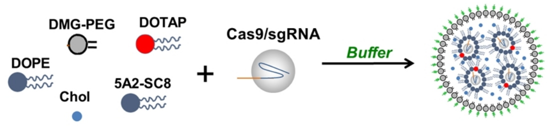

j

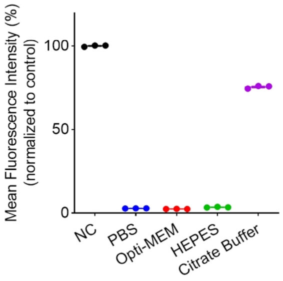

k

Buffer	Indels (%)
PBS	80.9
Opti-MEM	89.8
HEPES	75.6
Citrate Buffer	7.7

Supplementary Figure 6. The generalizable RNP delivery strategy is universal for ionizable cationic lipid nanoparticles (DLNPs, LLNPs, SNALPs), other cationic lipids that are positively charged at pH 7.4, and other neutral buffers. a, Scheme of LNP formulation with different ionizable lipids. **b,** Details of LNP formulations with different ionizable lipids, including determinate molar ratio and percentage of each component, and the weight ratio of total lipids to sgRNA. **c,** Chemical structures of ionizable cationic lipids used in formulations, including 5A2-SC8, C12-200, and Dlin-MC3-DMA. **d,** Mean Fluorescence Intensity (%) of HeLa-GFP cells following treatment with Cas9/sgGFP RNPs encapsulated in 5A2-DOT-10, C12-200-DOT-10, and MC3-DOT-10. The GFP fluorescence significantly decreased after treatment with all three formulations. **e,** Scheme of LNP formulation preparation with different permanently cationic lipids. **f,** Details of LNP formulations with different permanently cationic lipids, including determinate molar ratio and

percentage of each component, and the weight ratio of total lipids to sgRNA. **g,** Chemical structures of permanently cationic lipids used in formulations, including DOTAP, DDAB, and EPC. **h,** Mean Fluorescence Intensity (%) of HeLa-GFP cells after treatment with Cas9/sgGFP RNPs encapsulated in 5A2-DOT-10, 5A2-DDAB-10, and 5A2-EPC-10. Instead of DOTAP, other cationic lipids (DDAB and EPC) were also able to achieve efficient gene editing. **i,** Scheme of LNP formulation in different buffers. **j,** Mean Fluorescence Intensity (%) of HeLa-GFP cells after treatment with 5A2-DOT-10 formulated using different buffers, including PBS, Opti-MEM, HEPES, and Citrate Buffer. Neutral buffer was required for RNP encapsulation and delivery. **k,** Indels (%) at GFP loci in genomic DNA isolated from HeLa-GFP cells after treatment with 5A2-DOT-10 Cas9/sgGFP LNPs prepared using different buffers were measured using Sanger sequencing and TIDE analysis. All neutral buffers showed high gene editing in cells, demonstrating the importance of neutral buffers in nanoparticle preparation. Please note that Fig. 2g and 2h have been reproduced above in Supplementary Figure 6 to assemble relevant data together for enhanced clarity. Data of **d**, **h** and **j** are presented as mean ± s.e.m. (n= 3 biologically independent samples).

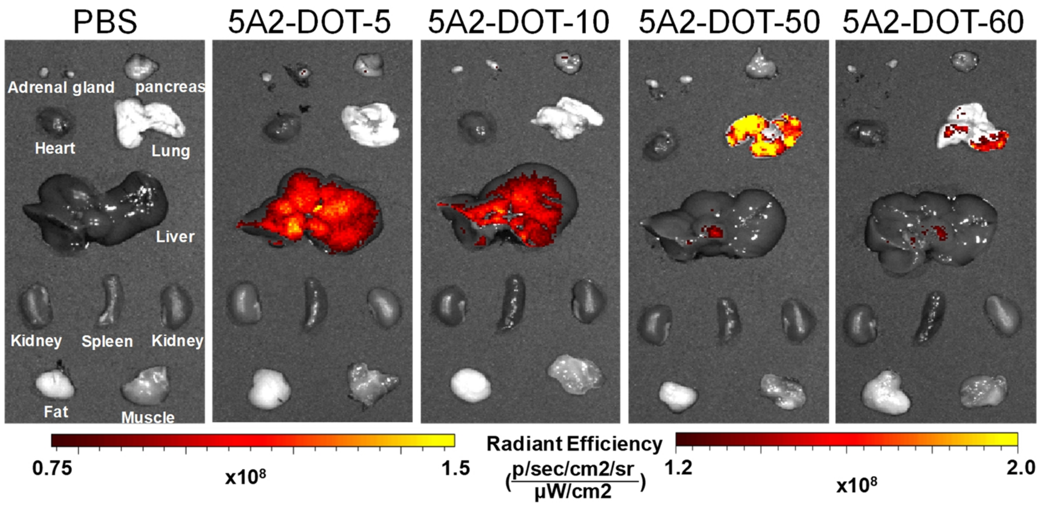

Supplementary Figure 7. Ex vivo images of td-Tomato mice treated with different 5A2-DOT-X formulations. With the increase of DOTAP level, the tissue specificity changed from liver to lung.

	ID (OT)	Sequence (20nt)	PAM	# mismatches	Locus
On-Target	sgPTEN	AGATCGTTAGCAGAAACAAA	AGG	0	chr19:32758464
Off-target (OT)	OT-1	AGCACGTTAGCAGAAACCAA	AGG	3	chr15:15308342
	OT-2	AGAATGTTAACAGAAACAAA	TGG	3	chr15:60681817
	OT-3	AGATTGTTATCACAAACAAA	TGG	3	chr6:74056110
	OT-4	AGATAGTTAGCACAAAGAAA	TGG	3	chr13:111927102
	OT-5	AGAGCGTTAGCAGACCCAAA	TGG	3	chr19:30941492
	OT-6	AGATGGTTAGCAGTAGCAAA	GGG	3	chrX:152078091
	OT-7	AGATGGCTAGCAGAAAAAAA	AGG	3	chr10:122894687
	OT-8	TGATCGTTAGCAGATACACA	TGG	3	chr2:26070703
	OT-9	AGAGTGTTAGCAGAAACAAT	TGG	3	chr11:22333524
	OT-10	AGATAGATAGCAGAAAGAAA	AGG	3	chr7:43650247

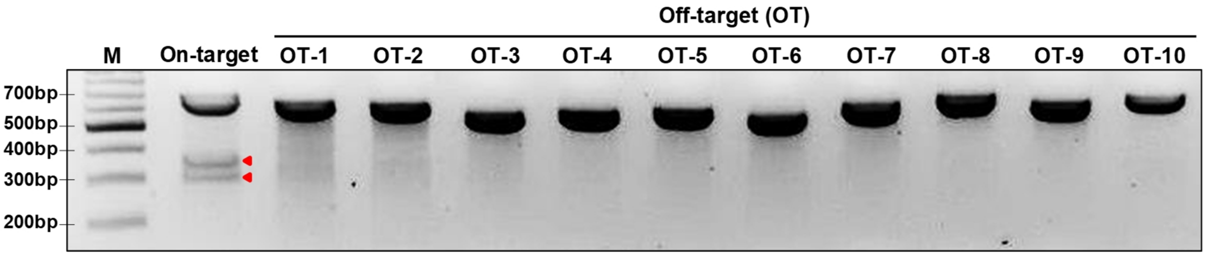

Supplementary Figure 8. **No off-target editing was detected after treatment with 5A2-DOT-50 nanoparticles targeting gene *PTEN*.** The top 10 potential off-target sites were amplified by PCR and analyzed by T7EI assay. On-target PCR products showed two clear cleavage bands (red arrows), but noting detected for all 10 potential off-target sites. Data was repeated three times independently with similar results.

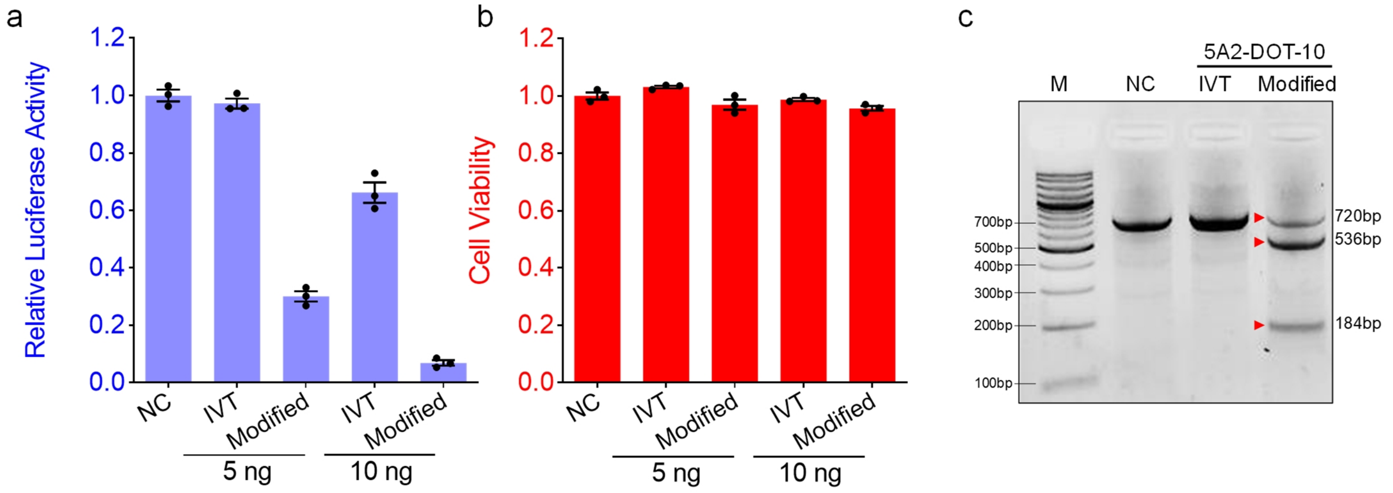

Supplementary Figure 9. Gene editing efficiency of unmodified sgRNA synthesized by IVT comparing to chemically modified and synthesized sgRNA. a, Relative luciferase activity in Hela-Luc-Cas9 cells after treatment with in vitro transcription (IVT) sgRNA and chemically modified sgRNA (2'-methyl 3'-phosphorothioate modifications in the first and last 3 nucleotides) encapsulated inside nanoparticles. Data are presented as mean ± s.e.m. (n= 3 biologically independent samples). **b,** Cell viabilities of Hela-Luc-Cas9 cells after treatment with IVT sgRNA and chemically modified sgRNA encapsulated inside nanoparticles. Data are presented as mean ± s.e.m. (n= 3 biologically independent samples). **c,** T7EI assay detecting gene editing efficiency of Cas9/IVT sgRNA and Cas9/chemically modified sgRNA encapsulated nanoparticles. Cleavage bands at 536 bp and 184 bp were observed clearly with modified sgRNA treatment group. Data was repeated three times independently with similar results.

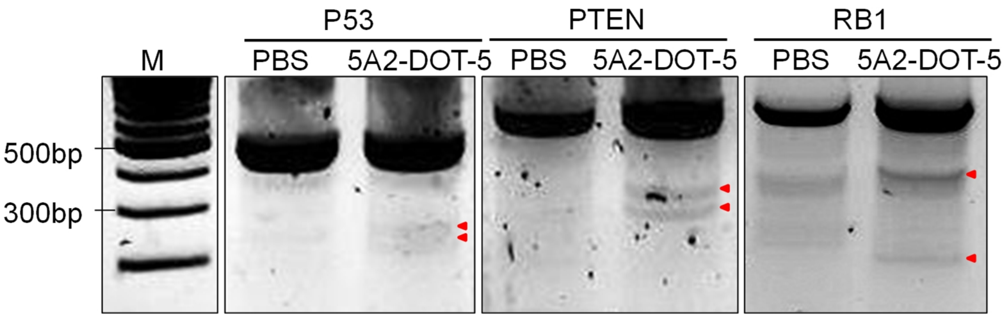

Supplementary Figure 10. Gene editing of P53, PTEN and RB1 genes in mouse liver after treatment with 5A2-DOT-5 LNPs encapsulating Cas9/sgP53/sgPTEN/sgRB1 RNPs. T7EI assay detecting the gene editing of liver genomic DNA at PTEN, P53 and RB1 genome loci, after treatment weekly for two weeks. PBS treatment group was used as control. Cleavage bands (red arrows) were detected at 261 bp and 215 bp of PCR amplicons targeting P53; cleavage bands (red arrows) were detected at 345 bp and 293 bp of PCR amplicons targeting PTEN; cleavage bands (red arrows) were detected at 395 bp and 207 bp of PCR amplicons targeting RB1. Data was repeated three times independently with similar results.

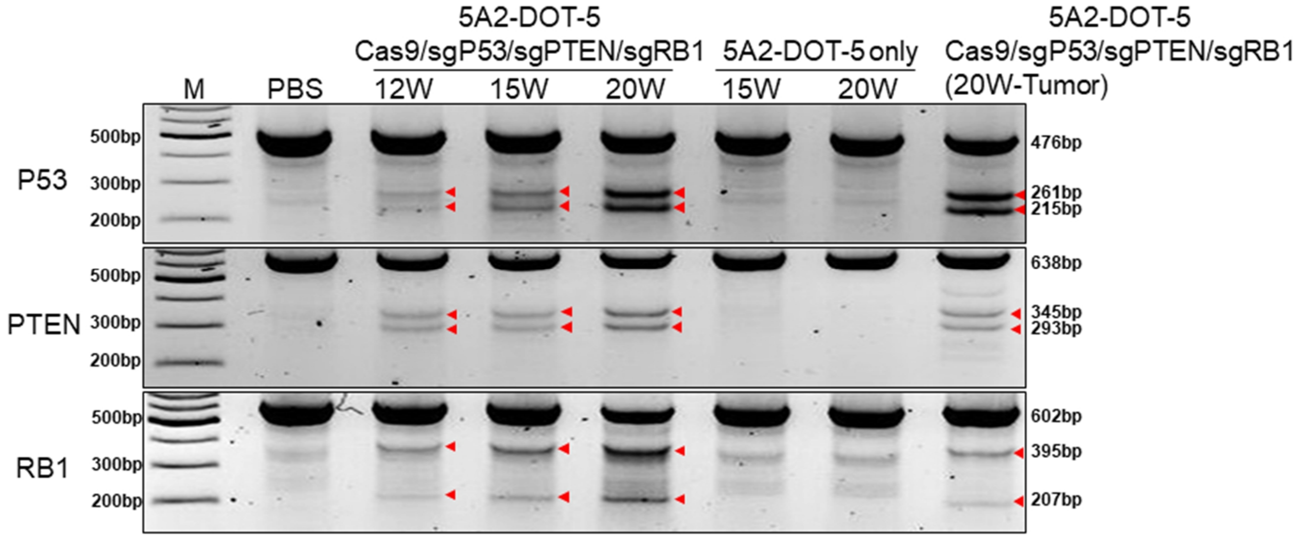

Supplementary Figure 11. T7EI assay detecting the gene editing of P53, PTEN and RB1 genes in mouse liver after treatment with 5A2-DOT-5 LNPs encapsulating Cas9/sgP53/sgPTEN/sgRB1 RNPs. PBS treatment and 5A2-DOT-5 only (no Cas9/sgRNA) treatment groups were used as controls. The T7EI result of

genome DNA extracted from mouse tumors after treatment with 5A2-DOT-5 LNPs encapsulating Cas9/sgP53/sgPTEN/sgRB1 RNPs for 20 weeks demonstrated the tumor generation was induced by knockout of these three genes, as cleavage bands (red arrows) were detected at all three genomic loci. Data was repeated three times independently with similar results.

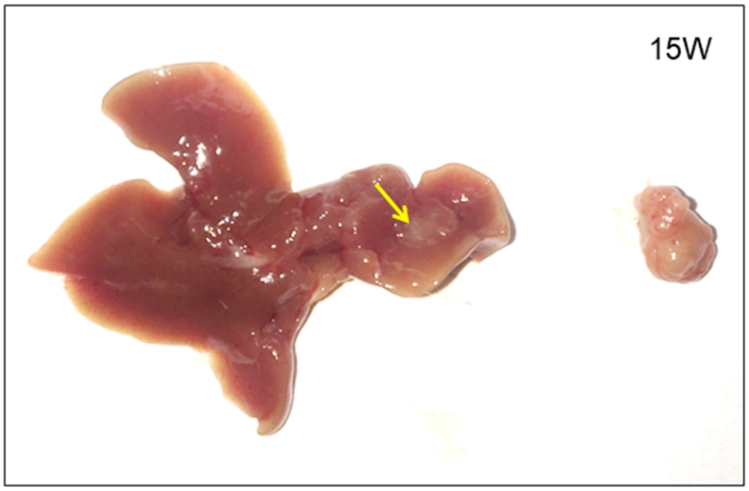

Supplementary Figure 12. Representative photograph of a mouse liver and excised tumors. The liver and tumor were excised from a mouse in the group treated with 5A2-DOT-5 LNPs encapsulating Cas9/sgP53/sgPTEN/sgRB1 RNPs for 15 weeks.

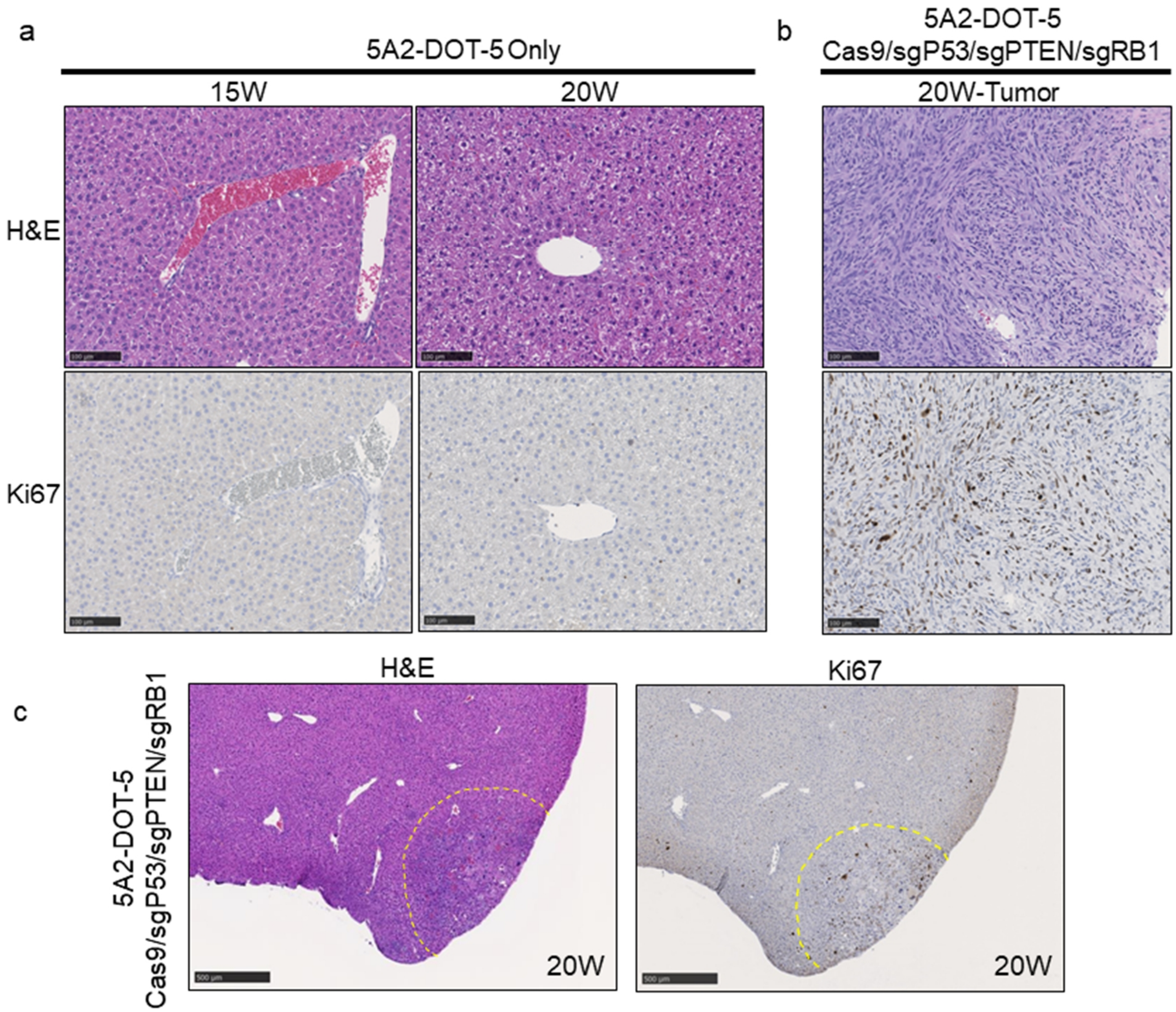

Supplementary Figure 13. Representative H&E and Ki67 staining images. Representative images from mouse livers following treatment with 5A2-DOT-5 LNPs only (no Cas9/sgRNA) (control) (time points of 15 weeks and 20 weeks) (**a**), tumors excised from a mouse in the group treated with 5A2-DOT-5 LNPs encapsulating Cas9/sgP53/sgPTEN/sgRB1 RNPs (time point of 20 weeks) (**b**). No morphological changes were detected with 5A2-DOT-5 LNPs only treatments, suggesting LNPs alone could not lead to tumors. Scale bar: 100 µm. **c,** Large view images of mouse liver tumor generation after treatment with 5A2-DOT-5 LNPs encapsulating Cas9/sgP53/sgPTEN/sgRB1 RNPs (time point of 20 weeks). Scale bar: 500 µm. Yellow dash circles showed the generation of liver tumor. Data was repeated three times independently with similar results.

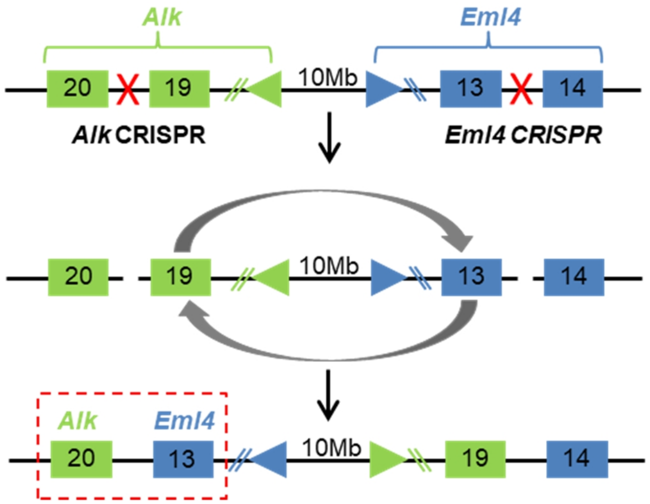

Supplementary Figure 14. Schematic presentation of Eml4-Alk rearrangement generated by gene editing at Eml4 and Alk loci simultaneously. Double DSBs generated at the intron 19 of Alk locus and the intron 13 of the Eml4 locus induced a chromosomal inversion and led to Eml4-Alk rearrangement in the chromosome.

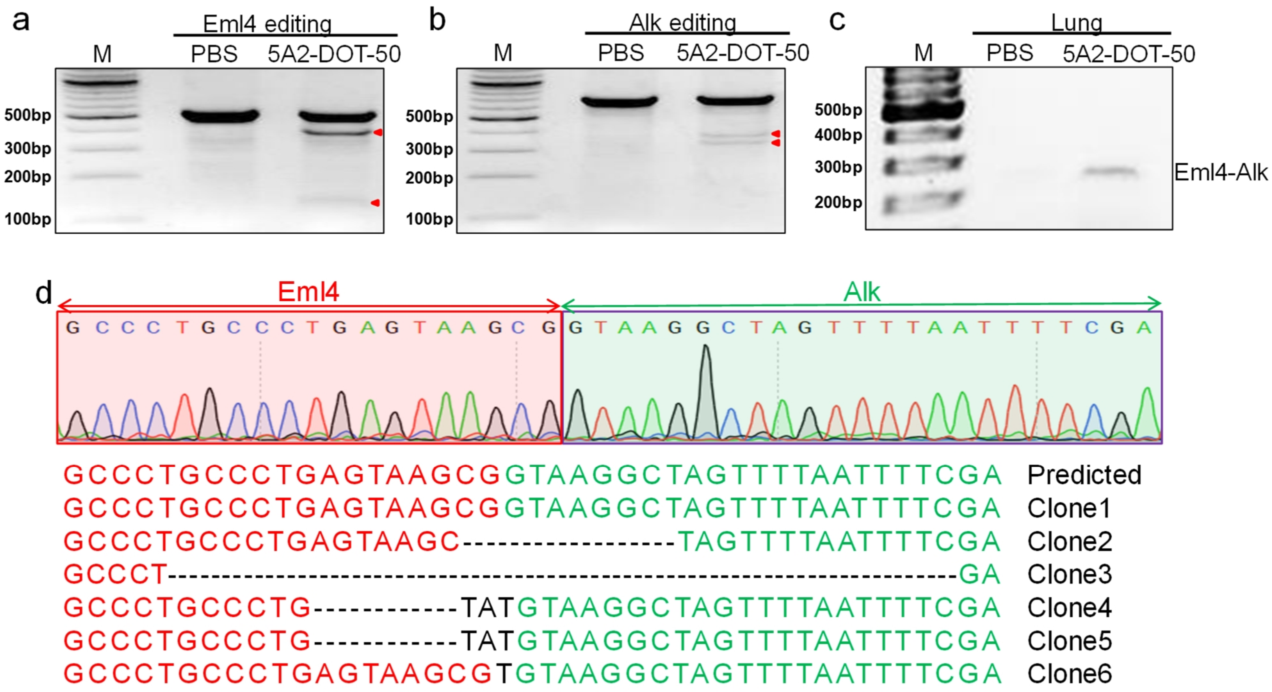

Supplementary Figure 15. Generation of Eml4-Alk rearrangements in mouse lungs. Mice were treated with 5A2-DOT-50 LNPs encapsulating Cas9/sgEml4/sgAlk RNPs (2 mg/kg total sgRNA) (time point of 7 days). Eml4 editing (**a**) and Alk editing (**b**) were detected in genomic DNA extracted from mouse lungs by the T7EI assay. **c,** PCR analysis was performed on genomic DNA extracted from mouse lungs to determine Eml4-Alk inversion. **d,** The PCR amplicons were sub-cloned and the sequences of 6 independent clones were listed, together with a representative chromatogram presented on the upper panel. The chromatogram was exactly the same as predicted for Eml4-Alk rearrangement. Data of **a**, **b**, **c** were repeated three times independently with similar results.

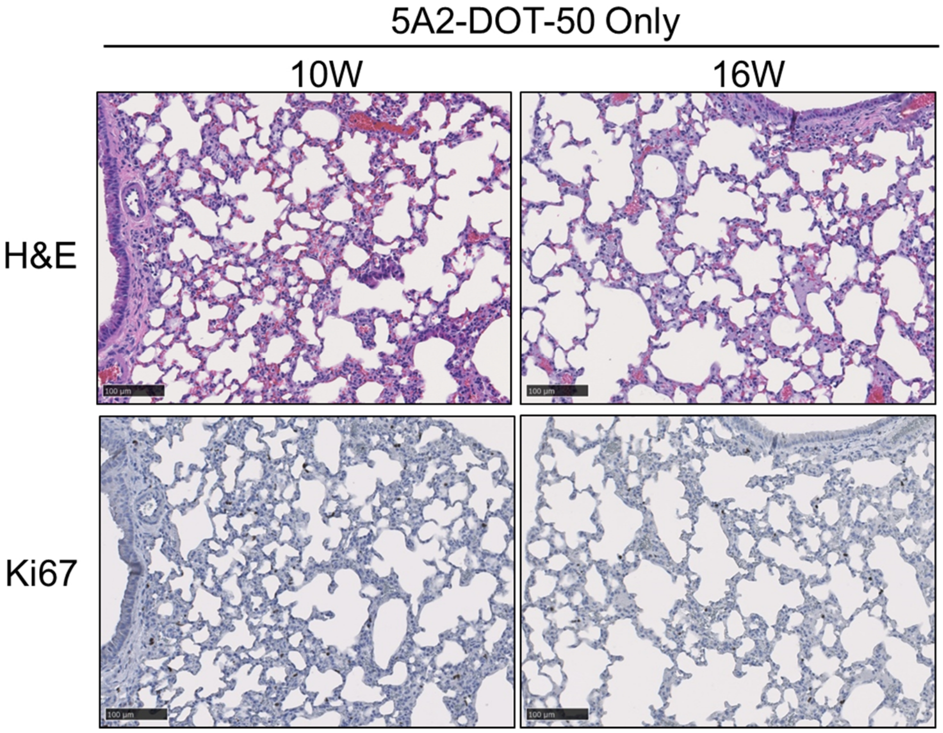

Supplementary Figure 16. H&E and Ki67 staining images of mouse livers treated with 5A2-DOT-50 LNPs Only (time points of 10 weeks and 16 weeks). The LNP dose equaled 1 mg/kg of total sgRNA. No morphological changes were detected in 5A2-DOT-50 LNPs Only (no Cas9/sgRNA) injected animals. Scale bar: 100µm. Data was repeated three times independently with similar results.

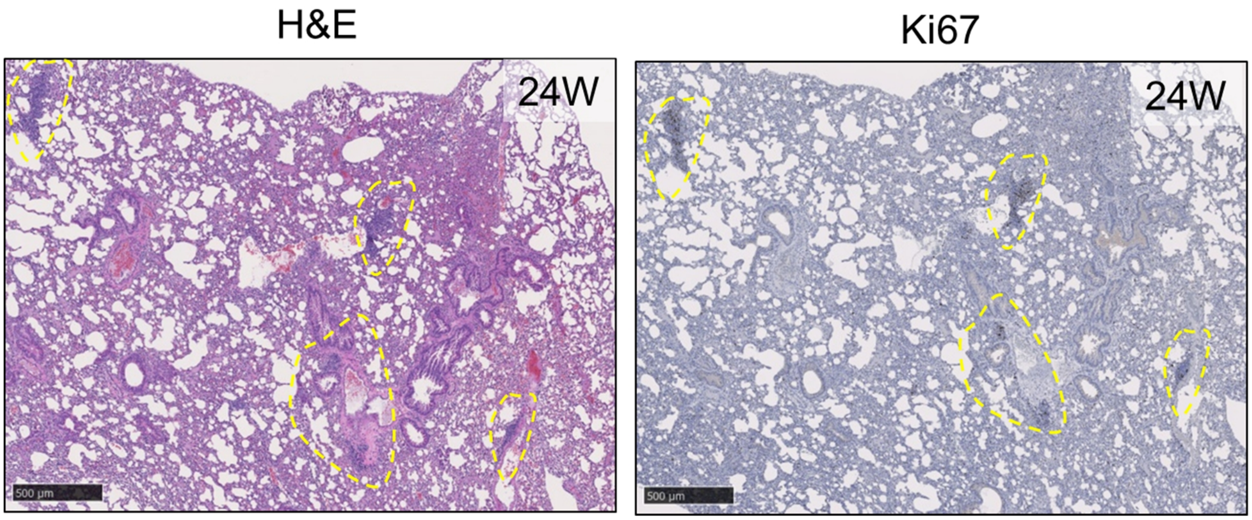

Supplementary Figure 17. Large view images of mouse lung after treatment with 5A2-DOT-50 LNPs encapsulating Cas9/sgEml4/sgAlk RNPs for 24 weeks. Scale bar: 500 μm. Several tumor lesions (highlighted with yellow dash circles) were observed in both H&E and Ki67 staining images. Data was repeated three times independently with similar results.

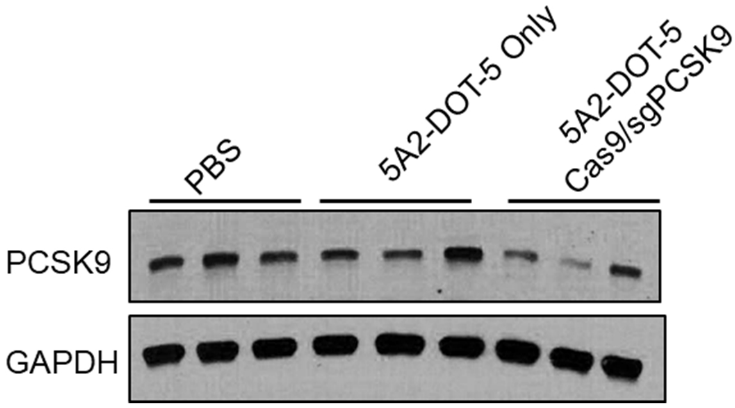

Supplementary Figure 18. Western blot analysis further demonstrated decreased expression of PCSK9 protein in mice livers. C57BL/6 mice were treated with 5A2-DOT-5 LNPs encapsulating Cas9/sgPCSK9 RNPs. PBS treatment group and nanoparticle only (5A2-DOT-5 Only) treatment groups were used as negative controls (n=3 biologically independent animals).

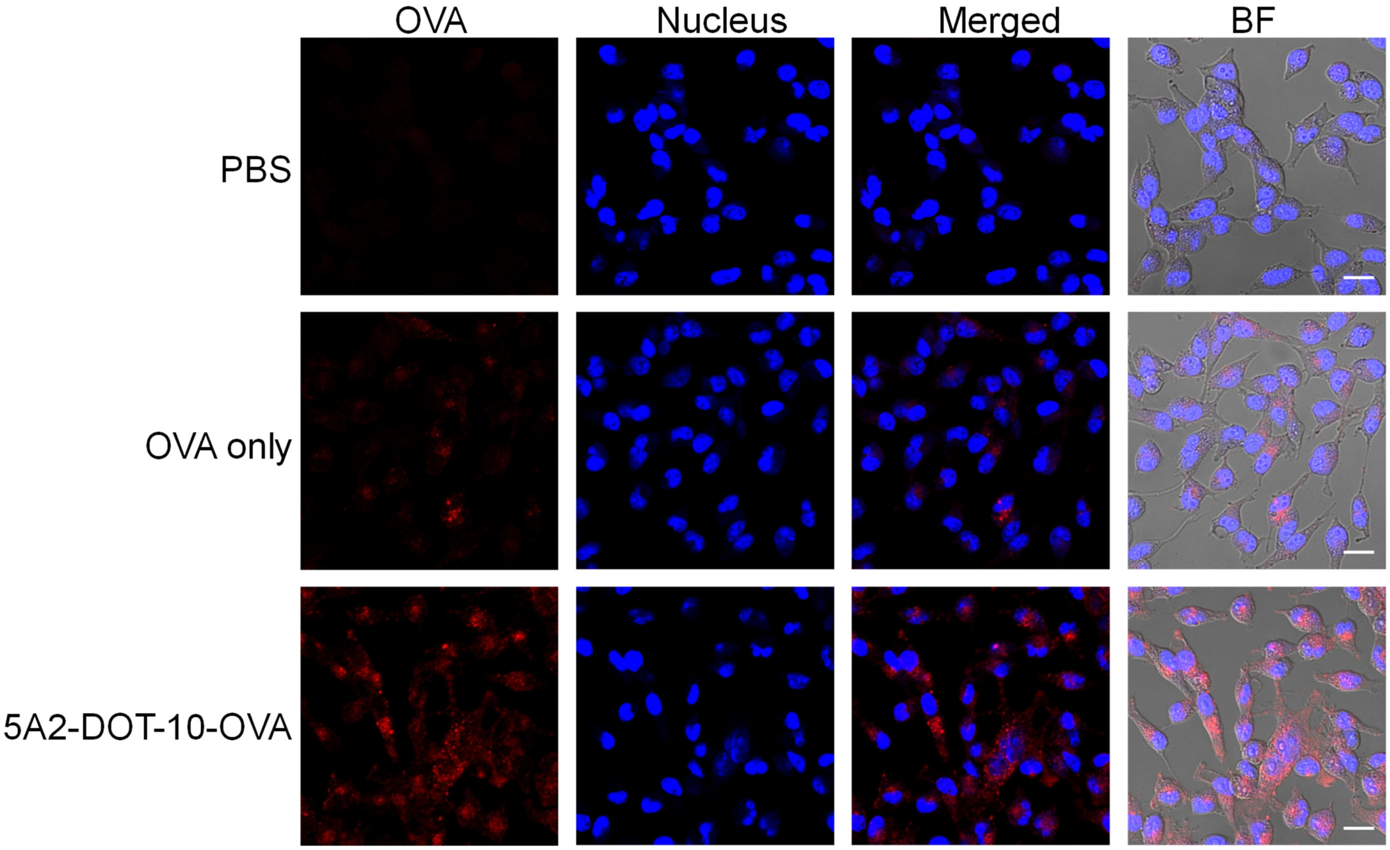

Supplementary Figure 19. 5A2-DOT-10 LNPs could deliver ovalbumin (OVA) protein efficiently into the cytoplasm of HeLa-Luc cells. Cells were treated with free rhodamine-labeled OVA protein and 5A2-DOT-10 LNPs encapsulating rhodamine-labeled OVA for 22 h before imaging by confocal microscopy (n=3 biologically independent samples). Scale bar: 20 µm.

Supplementary Figure 20. Uncropped original gel scans

Fig. 3h (T7EI for PTEN)

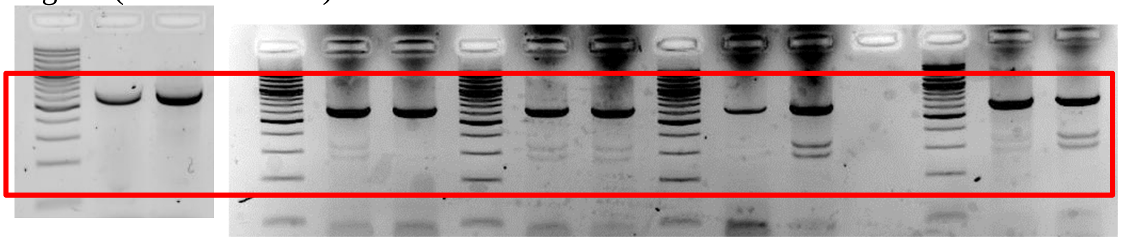

Fig. 3j (T7EI for P53, PTEN, Eml4, ALK, and RB1)

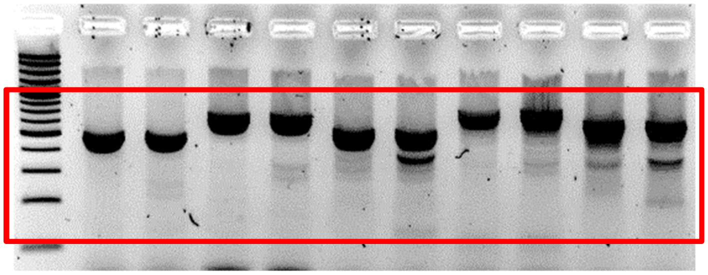

Fig. 4f (T7EI for Eml4 and Alk)

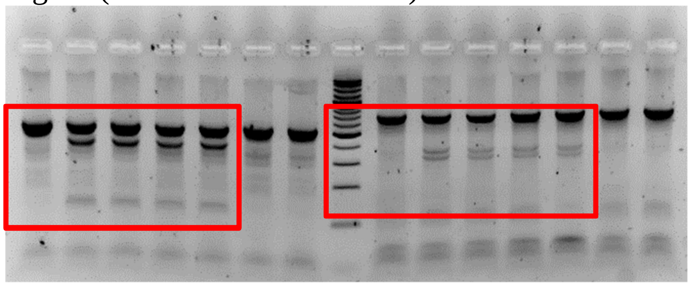

Fig. 4g (PCR for E/A and GAPDH)

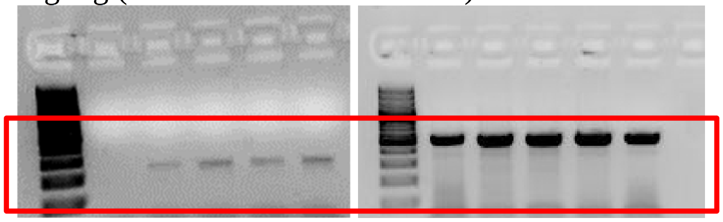

Fig. 5c (western blots for Dystrophin and Vinculin)

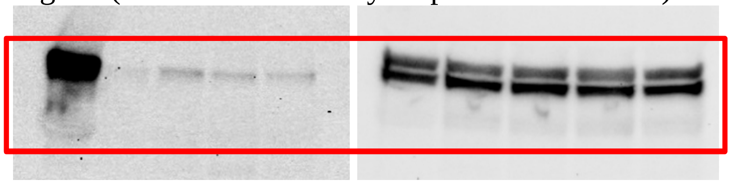

Fig. 5f (T7EI for PCSK9)

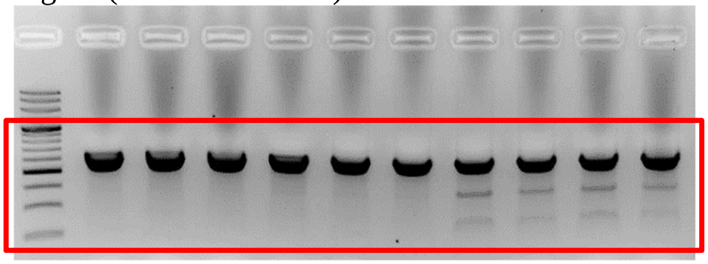

Supplementary Fig. 1 (T7EI for Luc)

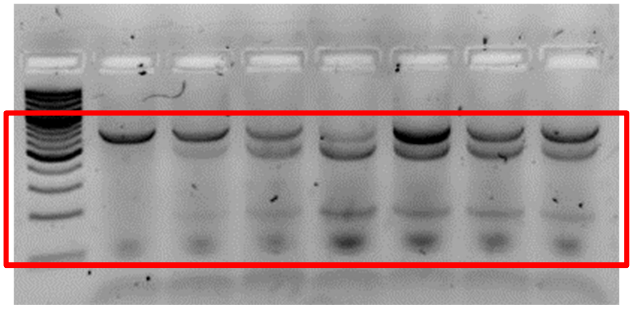

Supplementary Fig. 8 (T7EI for off-target testing)

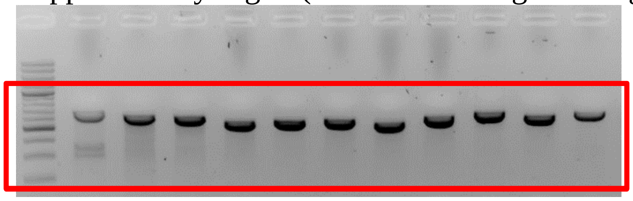

Supplementary Fig. 10 (T7EI for P53, PTEN and RB1)

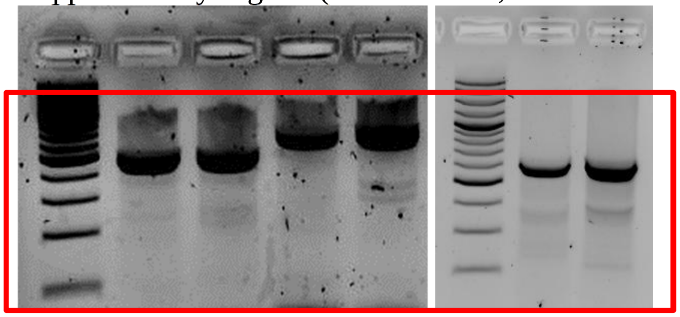

Supplementary Fig 11 (including Fig. 4b, T7EI for P53, PTEN and RB1)

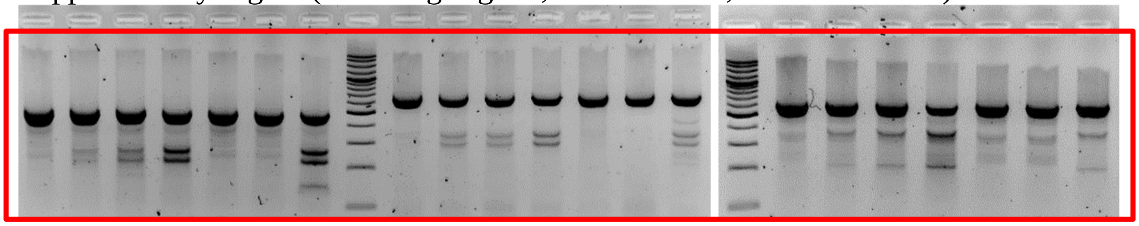

Supplementary Fig. 15 (T7EI for Eml4 and Alk, PCR for Eml4-Alk)

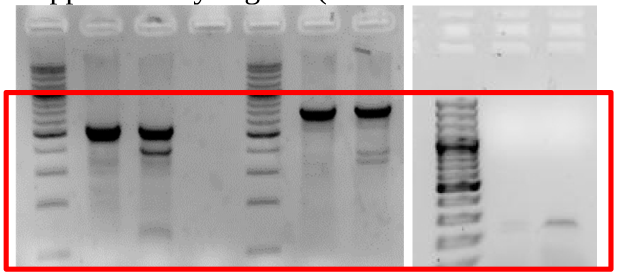

Supplementary Fig. 18. (Western blots for PCSK9 and GAPDH)

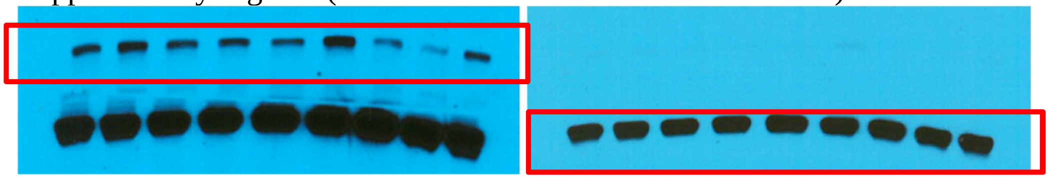

Supplementary Tables

Supplementary Table 1. Details of all sgRNA sequences used in this research.

Name	Target Sequences (5’ to 3’)	PAM (5’ to 3’)
sgLUC	CTTCGAAATGTCCGTTCGGT	TGG
sgGFP	GAAGTTCGAGGGCGACACCC	TGG
sgTOM	AAGTAAAACCTCTACAAATG	TGG
sgPTEN	AGATCGTTAGCAGAAACAAA	AGG
sgP53	GTGTAATAGCTCCTGCATGG	GGG
sgRB1	TCTTACCAGGATTCCATCCA	CGG
sgEml4	CCTGCCCTGAGTAAGCGACA	CGG
sgAlk	TCCTGGCATGTCTATCTGTA	AGG
sgDMD	CTTACAGGAACTCCAGGA	TGG
sgPCSK9	GCCCCATGTGGAGTACATTG	AGG

Supplementary Table 2. Details of primers used in this research.

Name	Forward Primers (5’ to 3’)	Reverse Primers (5’ to 3’)
LUC	ATGGAAGACGCCAAAAACATAAAGA AAGGCCCGGCGCCATTC	AACACTTAAAATCGCAGTATCCGGAATG
GFP	GTGGTGCCCATCCTGGTCGAG	CGCTTCTCGTTGGGGTCTTTGC
PTEN	ATCCGTCTTCTCCCCATTCCG	GACGAGCTCGCTAATCCAGTG
P53	ATAGAGACGCTGAGTCCGGTTC	CCTAAGCCCAAGAGGAAACAGA
RB1	CTGTGCTGGTGTGTGCAAACTATA	CTGTCACAGTGAAACTCGTTACTTTGTAT ATC
Eml4	ACAAGGCTCTGGCTTCCATTG	GATCAAAGCAAGGCCTTGTGCAT
Alk	TCTGAGCCCCTTCCATCTGACC	AGCTCAGCAGAAGCTCAGCAG
Eml4-Alk inversion (1st round)	CCCAGTCATCAGTTGCTATGCAATT	GGGTTTCCTTTGGTTCACAGATCCA

Eml4-Alk inversion (2nd round)	CGTTTTTCCACAAGAGCTAAGGCT	GTGGTTTGGTCACATCTCAGGTG
GAPDH	CACGTAGCTCAGGCCTCTGC	CGGGTCAGCAAGACCTGC
PCSK9	CCATGGCCAGCACTGGTATCC	CTCTTGCATTTCTGCATGGAGCAATG
OT-1	GCTTCACTGGGTTTGAAAGTTCCC	TCCAAGAAGCATGGAGTTAATGAGACAAA
OT-2	CATATGTAATCGAGATGAATTTACACTGCCT	CCCAAGATTAGGGAGATGATTCCTCAC
OT-3	CTATGCATCCCCTTTTCAGACACACA	TCATGACAACTATGTAGTAACCAACCCACTT
OT-4	CTGACTGGCTTATGCTGGAGAG	CCACTCTGCAGCTGATATTAAATAGCT
OT-5	AGTTGCTCAGTGACATGCCTTACT	TGAGCAAACCTCCAAACACTCAAAGT
OT-6	CAGCACCAGCTCTAGATATAGGTAGGT	TTAGATGTTACACAGCCACTAGAATTCATTCC
OT-7	TACCGAATCTTGTGTGAGTGTGCATTTG	AGGAGGGAGGAGAAAGCCACAG
OT-8	TCAGAGTCTGCGGGACAGTTCT	CAGAAGAGGGCATCAGATCCCATTACA
OT-9	TGACAAGACACACTTGGGTGGTAGTG	TCACCGTACATTTGAATTACAGGACCTG
OT-10	CCGAGTTAAGGCACGTTTGG	CATGGCGAGGAAGGCAACATG
