# Supplementary material for: Systemic nanoparticle delivery of CRISPR-Cas9 ribonucleoproteins for effective tissue specific genome editing
Source: Nat Commun. 2020 Jun 26;11:3232. doi: 10.1038/s41467-020-17029-3 (PMC7320157; doi:10.1038/s41467-020-17029-3)
Supplement: Supplementary file 2 — Reporting Summary [file 41467_2020_17029_MOESM2_ESM.pdf]

nature research

Corresponding author(s): Daniel J. Siegwart

Last updated by author(s): May 11, 2020

Reporting Summary

Nature Research wishes to improve the reproducibility of the work that we publish. This form provides structure for consistency and transparency in reporting. For further information on Nature Research policies, see Authors & Referees and the Editorial Policy Checklist .

Statistics

For all statistical analyses, confirm that the following items are present in the figure legend, table legend, main text, or Methods section.

n/a	Confirmed	
☐	☒	The exact sample size (*n*) for each experimental group/condition, given as a discrete number and unit of measurement
☐	☒	A statement on whether measurements were taken from distinct samples or whether the same sample was measured repeatedly
☐	☒	The statistical test(s) used AND whether they are one- or two-sided<br>*Only common tests should be described solely by name; describe more complex techniques in the Methods section.*
☒	☐	A description of all covariates tested
☒	☐	A description of any assumptions or corrections, such as tests of normality and adjustment for multiple comparisons
☐	☒	A full description of the statistical parameters including central tendency (e.g. means) or other basic estimates (e.g. regression coefficient) AND variation (e.g. standard deviation) or associated estimates of uncertainty (e.g. confidence intervals)
☐	☒	For null hypothesis testing, the test statistic (e.g. *F*, *t*, *r*) with confidence intervals, effect sizes, degrees of freedom and *P* value noted<br>*Give P values as exact values whenever suitable.*
☒	☐	For Bayesian analysis, information on the choice of priors and Markov chain Monte Carlo settings
☒	☐	For hierarchical and complex designs, identification of the appropriate level for tests and full reporting of outcomes
☒	☐	Estimates of effect sizes (e.g. Cohen's *d*, Pearson's *r*), indicating how they were calculated

Our web collection on statistics for biologists contains articles on many of the points above.

Software and code

Policy information about availability of computer code

Data collection	Zetasizer software version 7.13 Malvern Panalytical<br>ZEN x64 software version 1.1.0 (Carl Zeiss Microscopy GmbH)<br>BZ-X Viewer software version 1.0.0 (KEYENCE Corporation)<br>Living Image software version 4.4.0.0 (64-bit, Caliper Life Sciences)<br>BD FACSDiva software version 8.0.1 (BD LSRFortessa)<br>NDP.scan software version3.1.9 (NANOZOOMER, Hamamatsu)<br>Cas-OFFinder online webtool (http://www.rgenome.net/cas-offinder/)
Data analysis	Image J 1.50b Java 1.8.0_60 (64-bit) (National Institutes of Health, USA)<br>FLOWJO software version 7.6 (FLOWJO)<br>ZEN 2010 software version 6.0.62 (Carl Zeiss MicroImaging GmbH)<br>NDP.view 2 software version 2.7.25 (Hamamatsu)<br>BZ-X Analyzer software version 1.0.0 (KEYENCE Corporation)<br>GraphPad Prism 7 software version 7.04 (GraphPad Software)<br>Living Image software version 4.4.0.0 (64-bit, Caliper Life Sciences)<br>TIDE Analysis tool (no version information available), (analyzing indels by decomposition): https://tide.deskgen.com/

For manuscripts utilizing custom algorithms or software that are central to the research but not yet described in published literature, software must be made available to editors/reviewers. We strongly encourage code deposition in a community repository (e.g. GitHub). See the Nature Research guidelines for submitting code & software for further information.

Data

Policy information about availability of data

All manuscripts must include a data availability statement. This statement should provide the following information, where applicable:

- Accession codes, unique identifiers, or web links for publicly available datasets
- A list of figures that have associated raw data
- A description of any restrictions on data availability

The authors declare that all data supporting the findings of this study are available within the paper [and its supplementary information files].
The data that support the findings of this study are available from the corresponding author upon reasonable request.

Field-specific reporting

Please select the one below that is the best fit for your research. If you are not sure, read the appropriate sections before making your selection.

[x] Life sciences   [ ] Behavioural & social sciences   [ ] Ecological, evolutionary & environmental sciences

For a reference copy of the document with all sections, see nature.com/documents/nr-reporting-summary-flat.pdf

Life sciences study design

All studies must disclose on these points even when the disclosure is negative.

Sample size	Following standards of the field, including power analysis, sample sizes were estimated which were capable of yielding statistically significant differences.
Data exclusions	No data were excluded from the analyses.
Replication	Reported results were consistently replicated across multiple experiments with all replicates generating similar results. We have used tdTomato reporter mice (Ai9 mice) and C57BL/6 mice to confirm tissue specific gene editing in liver and lung. The successful liver tumor mice model and lung tumor mice model creations also confirmed that our experimental finding is reproducible. Some key data generated by one co-author were repeated by other co-authors.
Randomization	No randomization was necessary for cell based experiments because investigators were comparing nanoformulations under well controlled conditions. For animal experiments, mice with ages of 8-10 weeks were randomly allocated into each treatment group.
Blinding	Due to the proof of concept nature of this study, true blinding of experiments was not possible. However, some of experiments collection and analyses were conducted by separate individuals. These collectors/analyzers were not aware which samples corresponded to which experimental groups at the time of data collection and analysis. For example, Flow Cytometry data and Sequencing data were collected and analyzed in this manner wherever possible.

Behavioural & social sciences study design

All studies must disclose on these points even when the disclosure is negative.

Study description	*Briefly describe the study type including whether data are quantitative, qualitative, or mixed-methods (e.g. qualitative cross-sectional, quantitative experimental, mixed-methods case study).*
Research sample	*State the research sample (e.g. Harvard university undergraduates, villagers in rural India) and provide relevant demographic information (e.g. age, sex) and indicate whether the sample is representative. Provide a rationale for the study sample chosen. For studies involving existing datasets, please describe the dataset and source.*
Sampling strategy	*Describe the sampling procedure (e.g. random, snowball, stratified, convenience). Describe the statistical methods that were used to predetermine sample size OR if no sample-size calculation was performed, describe how sample sizes were chosen and provide a rationale for why these sample sizes are sufficient. For qualitative data, please indicate whether data saturation was considered, and what criteria were used to decide that no further sampling was needed.*
Data collection	*Provide details about the data collection procedure, including the instruments or devices used to record the data (e.g. pen and paper, computer, eye tracker, video or audio equipment) whether anyone was present besides the participant(s) and the researcher, and whether the researcher was blind to experimental condition and/or the study hypothesis during data collection.*
Timing	*Indicate the start and stop dates of data collection. If there is a gap between collection periods, state the dates for each sample cohort.*
Data exclusions	*If no data were excluded from the analyses, state so OR if data were excluded, provide the exact number of exclusions and the rationale behind them, indicating whether exclusion criteria were pre-established.*

Non-participation	*State how many participants dropped out/declined participation and the reason(s) given OR provide response rate OR state that no participants dropped out/declined participation.*
Randomization	*If participants were not allocated into experimental groups, state so OR describe how participants were allocated to groups, and if allocation was not random, describe how covariates were controlled.*

Ecological, evolutionary & environmental sciences study design

All studies must disclose on these points even when the disclosure is negative.

Study description	*Briefly describe the study. For quantitative data include treatment factors and interactions, design structure (e.g. factorial, nested, hierarchical), nature and number of experimental units and replicates.*
Research sample	*Describe the research sample (e.g. a group of tagged Passer domesticus, all Stenocereus thurberi within Organ Pipe Cactus National Monument), and provide a rationale for the sample choice. When relevant, describe the organism taxa, source, sex, age range and any manipulations. State what population the sample is meant to represent when applicable. For studies involving existing datasets, describe the data and its source.*
Sampling strategy	*Note the sampling procedure. Describe the statistical methods that were used to predetermine sample size OR if no sample-size calculation was performed, describe how sample sizes were chosen and provide a rationale for why these sample sizes are sufficient.*
Data collection	*Describe the data collection procedure, including who recorded the data and how.*
Timing and spatial scale	*Indicate the start and stop dates of data collection, noting the frequency and periodicity of sampling and providing a rationale for these choices. If there is a gap between collection periods, state the dates for each sample cohort. Specify the spatial scale from which the data are taken*
Data exclusions	*If no data were excluded from the analyses, state so OR if data were excluded, describe the exclusions and the rationale behind them, indicating whether exclusion criteria were pre-established.*
Reproducibility	*Describe the measures taken to verify the reproducibility of experimental findings. For each experiment, note whether any attempts to repeat the experiment failed OR state that all attempts to repeat the experiment were successful.*
Randomization	*Describe how samples/organisms/participants were allocated into groups. If allocation was not random, describe how covariates were controlled. If this is not relevant to your study, explain why.*
Blinding	*Describe the extent of blinding used during data acquisition and analysis. If blinding was not possible, describe why OR explain why blinding was not relevant to your study.*

Did the study involve field work? ☐ Yes ☐ No

Field work, collection and transport

Field conditions	*Describe the study conditions for field work, providing relevant parameters (e.g. temperature, rainfall).*
Location	*State the location of the sampling or experiment, providing relevant parameters (e.g. latitude and longitude, elevation, water depth).*
Access and import/export	*Describe the efforts you have made to access habitats and to collect and import/export your samples in a responsible manner and in compliance with local, national and international laws, noting any permits that were obtained (give the name of the issuing authority, the date of issue, and any identifying information).*
Disturbance	*Describe any disturbance caused by the study and how it was minimized.*

Reporting for specific materials, systems and methods

We require information from authors about some types of materials, experimental systems and methods used in many studies. Here, indicate whether each material, system or method listed is relevant to your study. If you are not sure if a list item applies to your research, read the appropriate section before selecting a response.

Materials & experimental systems

n/a	Involved in the study
☐	☒ Antibodies
☐	☒ Eukaryotic cell lines
☒	☐ Palaeontology
☐	☒ Animals and other organisms
☒	☐ Human research participants
☒	☐ Clinical data

Methods

n/a	Involved in the study
☒	☐ ChIP-seq
☐	☒ Flow cytometry
☒	☐ MRI-based neuroimaging

Antibodies

Antibodies used

1. We used Ki-67 monoclonal antibody in the Immunohistochemistry (IHC) study to determine tumor proliferation in Figure 4D, 4I and SI figures. Supplier name: ThermoFisher Scientific (Invitrogen); catalog number: MA5-14520;clone name: SP6;
2. We used monoclonal anti-dystrophin antibody in the Immunofluorescence (IF) imaging (Figure 4K) and Western Blot (WB) study (Figure 4L) to determine dystrophin expression in TA muscles. Supplier name: Sigma-Aldrich; catalog number: D8168;clone name: MANDYS8;
3. We used monoclonal anti-vinculin antibody in the WB study (Figure 4L) to determine vinculin expression in TA muscles, as a loading control. Supplier name: Sigma-Aldrich; catalog number: V9131;clone name: hVIN-1;

Validation

1.The Ki-67 antibody has been validated to be used for IHC at dilution of 1:200 from the manufacturer's website and it is also mentioned species reactivity with mouse. (https://www.thermofisher.com/antibody/product/Ki-67-Antibody-clone-SP6-Monoclonal/MA5-14520). Our data provided also confirmed that it can be applied to IHC study of mouse tissues.
2.The anti-dystrophin antibody has been validated to be used for IF study (dilution at 1:1000) and WB study (dilution at 1:1000) from the manufacturer's website. It is also mentioned species reactivity with mouse. (https://www.sigmaaldrich.com/catalog/product/sigma/d8168?lang=en&region=US). Our data provided in the Figure 4K and 4L also validated that it can be applied to IF and WB studies of mouse tissues.
3.The anti-vinculin antibody has been validated to be used for WB study (dilution at 1:5000) from the manufacturer's website. It is also mentioned species reactivity with mouse. (https://www.sigmaaldrich.com/catalog/product/sigma/v9131?lang=en&region=US). Our data provided in the Figure 4L further validated that it can be applied to WB studies of mouse tissues.

Eukaryotic cell lines

Policy information about cell lines

Cell line source(s)

HeLa cells were originally obtained from ATTC. Derived HeLa-Luc and HeLa-GFP reporter cells were generated using lentiviruses from HeLa cells originally obtained from ATTC.

Authentication

The cell lines were not further authenticated after receiving from ATTC.

Mycoplasma contamination

The cell lines were not tested for mycoplasma contamination.

Commonly misidentified lines
(See ICLAC register)

No commonly misidentified cell lines were used.

Palaeontology

Specimen provenance

Provide provenance information for specimens and describe permits that were obtained for the work (including the name of the issuing authority, the date of issue, and any identifying information).

Specimen deposition

Indicate where the specimens have been deposited to permit free access by other researchers.

Dating methods

If new dates are provided, describe how they were obtained (e.g. collection, storage, sample pretreatment and measurement), where they were obtained (i.e. lab name), the calibration program and the protocol for quality assurance OR state that no new dates are provided.

☐ Tick this box to confirm that the raw and calibrated dates are available in the paper or in Supplementary Information.

Animals and other organisms

Policy information about studies involving animals; ARRIVE guidelines recommended for reporting animal research

Laboratory animals

C57BL/6 mice were obtained from the UTSW Mouse Breeding Core Facility. Male or female mice with age of 8-10 weeks were used. B6.Cg-Gt(ROSA)26Sortm9(CAGtdTomato)Hze/J mice (also known as Ai9 or Ai9(RCL-tdT) mice) were obtained from The Jackson Laboratory (007909) and bred to maintain homozygous expression of the Cre reporter allele that has a loxP-flanked STOP cassette preventing transcription of a CAG promoter-driven red fluorescent tdTomato protein. Following Cre-mediated

	recombination or CRISPR/Cas9 based gene editing, Ai9 mice will express tdTomato fluorescence. Ai9 mice are congenic on the C57BL/6J genetic background. Ai9 female mice with ages of 8-10 weeks were used. DMD mouse models were provided by our collaborator Prof. Eric N. Olson's lab. Male ΔEx44 DMD mice with age of P12 were used.
Wild animals	The study did not involve wild animals.
Field-collected samples	The study did not involve samples collected from the field.
Ethics oversight	All animal experiments were approved by the Institution Animal Care and Use Committees of The University of Texas Southwestern Medical Center and were consistent with local, state and federal regulations as applicable.

Note that full information on the approval of the study protocol must also be provided in the manuscript.

Human research participants

Policy information about studies involving human research participants

Population characteristics	*Describe the covariate-relevant population characteristics of the human research participants (e.g. age, gender, genotypic information, past and current diagnosis and treatment categories). If you filled out the behavioural & social sciences study design questions and have nothing to add here, write "See above."*
Recruitment	*Describe how participants were recruited. Outline any potential self-selection bias or other biases that may be present and how these are likely to impact results.*
Ethics oversight	*Identify the organization(s) that approved the study protocol.*

Note that full information on the approval of the study protocol must also be provided in the manuscript.

Clinical data

Policy information about clinical studies

All manuscripts should comply with the ICMJEguidelines for publication of clinical research and a completedCONSORT checklist must be included with all submissions.

Clinical trial registration	*Provide the trial registration number from ClinicalTrials.gov or an equivalent agency.*
Study protocol	*Note where the full trial protocol can be accessed OR if not available, explain why.*
Data collection	*Describe the settings and locales of data collection, noting the time periods of recruitment and data collection.*
Outcomes	*Describe how you pre-defined primary and secondary outcome measures and how you assessed these measures.*

ChIP-seq

Data deposition

☐ Confirm that both raw and final processed data have been deposited in a public database such as GEO.

☐ Confirm that you have deposited or provided access to graph files (e.g. BED files) for the called peaks.

Data access links<br>*May remain private before publication.*	*For "Initial submission" or "Revised version" documents, provide reviewer access links. For your "Final submission" document, provide a link to the deposited data.*
Files in database submission	*Provide a list of all files available in the database submission.*
Genome browser session<br>(e.g. UCSC)	*Provide a link to an anonymized genome browser session for "Initial submission" and "Revised version" documents only, to enable peer review. Write "no longer applicable" for "Final submission" documents.*

Methodology

Replicates	*Describe the experimental replicates, specifying number, type and replicate agreement.*
Sequencing depth	*Describe the sequencing depth for each experiment, providing the total number of reads, uniquely mapped reads, length of reads and whether they were paired- or single-end.*
Antibodies	*Describe the antibodies used for the ChIP-seq experiments; as applicable, provide supplier name, catalog number, clone name, and lot number.*
Peak calling parameters	*Specify the command line program and parameters used for read mapping and peak calling, including the ChIP, control and index files used.*
Data quality	*Describe the methods used to ensure data quality in full detail, including how many peaks are at FDR 5% and above 5-fold enrichment.*

Software

Describe the software used to collect and analyze the ChIP-seq data. For custom code that has been deposited into a community repository, provide accession details.

Flow Cytometry

Plots

Confirm that:

- [ ] The axis labels state the marker and fluorochrome used (e.g. CD4-FITC).
- [ ] The axis scales are clearly visible. Include numbers along axes only for bottom left plot of group (a 'group' is an analysis of identical markers).
- [ ] All plots are contour plots with outliers or pseudocolor plots.
- [ ] A numerical value for number of cells or percentage (with statistics) is provided.

Methodology

Sample preparation

HeLa-GFP reporter cells were cultured in DMEM containing 10% FBS and 1% penicillin/streptomycin at 37 °C / 5% CO2. For the experiments, HeLa-GFP cells were seeded into 12-well plates at a cell density of 1.5×105 cells per well and incubated overnight. Then, the medium was replaced with 0.5 mL of fresh complete DMEM and 100 uL of nanoparticle dispersion were added (the final concentration of sgRNA was fixed at 24 nM). 1mL of fresh medium was added into each well at day 2 after treatments to maintain enough nutrition. Three days after treatment, the cells were collected, washed with PBS, re-suspended in PBS, and analyzed using a BD Analyzers LSRFortessa SORP (BD Biosciences).

Instrument

BD Analyzers LSRFortessa SORP (BD Biosciences)

Software

Data collection: BD FACSDiva software version 8.0.1 (BD LSRFortessa);
Data analysis: FLOWJO software version 7.6 (FLOWJO)

Cell population abundance

We used cell lines to evaluate GFP knockout efficiency via detecting the GFP mean fluorescence intensity.

Gating strategy

No cell exclusion was needed to determine the GFP mean fluorescence intensity..

- [ ] Tick this box to confirm that a figure exemplifying the gating strategy is provided in the Supplementary Information.

Magnetic resonance imaging

Experimental design

Design type

Indicate task or resting state; event-related or block design.

Design specifications

Specify the number of blocks, trials or experimental units per session and/or subject, and specify the length of each trial or block (if trials are blocked) and interval between trials.

Behavioral performance measures

State number and/or type of variables recorded (e.g. correct button press, response time) and what statistics were used to establish that the subjects were performing the task as expected (e.g. mean, range, and/or standard deviation across subjects).

Acquisition

Imaging type(s)

Specify: functional, structural, diffusion, perfusion.

Field strength

Specify in Tesla

Sequence & imaging parameters

Specify the pulse sequence type (gradient echo, spin echo, etc.), imaging type (EPI, spiral, etc.), field of view, matrix size, slice thickness, orientation and TE/TR/flip angle.

Area of acquisition

State whether a whole brain scan was used OR define the area of acquisition, describing how the region was determined.

Diffusion MRI ☐ Used ☐ Not used

Preprocessing

Preprocessing software

Provide detail on software version and revision number and on specific parameters (model/functions, brain extraction, segmentation, smoothing kernel size, etc.).

Normalization

If data were normalized/standardized, describe the approach(es): specify linear or non-linear and define image types used for transformation OR indicate that data were not normalized and explain rationale for lack of normalization.

Normalization template | *Describe the template used for normalization/transformation, specifying subject space or group standardized space (e.g. original Talairach, MNI305, ICBM152) OR indicate that the data were not normalized.*

Noise and artifact removal | *Describe your procedure(s) for artifact and structured noise removal, specifying motion parameters, tissue signals and physiological signals (heart rate, respiration).*

Volume censoring | *Define your software and/or method and criteria for volume censoring, and state the extent of such censoring.*

Statistical modeling & inference

Model type and settings | *Specify type (mass univariate, multivariate, RSA, predictive, etc.) and describe essential details of the model at the first and second levels (e.g. fixed, random or mixed effects; drift or auto-correlation).*

Effect(s) tested | *Define precise effect in terms of the task or stimulus conditions instead of psychological concepts and indicate whether ANOVA or factorial designs were used.*

Specify type of analysis: ☐ Whole brain ☐ ROI-based ☐ Both

Statistic type for inference
(See Eklund et al. 2016) | *Specify voxel-wise or cluster-wise and report all relevant parameters for cluster-wise methods.*

Correction | *Describe the type of correction and how it is obtained for multiple comparisons (e.g. FWE, FDR, permutation or Monte Carlo).*

Models & analysis

n/a	Involved in the study
☐	☐ Functional and/or effective connectivity
☐	☐ Graph analysis
☐	☐ Multivariate modeling or predictive analysis

Functional and/or effective connectivity | *Report the measures of dependence used and the model details (e.g. Pearson correlation, partial correlation, mutual information).*

Graph analysis | *Report the dependent variable and connectivity measure, specifying weighted graph or binarized graph, subject- or group-level, and the global and/or node summaries used (e.g. clustering coefficient, efficiency, etc.).*

Multivariate modeling and predictive analysis | *Specify independent variables, features extraction and dimension reduction, model, training and evaluation metrics.*
